# Risk factors for internalizing symptoms: The influence of empathy, theory of mind, and negative thinking processes

**DOI:** 10.1002/hbm.26576

**Published:** 2024-02-24

**Authors:** Annika C. Konrad, Katharina Förster, Jason Stretton, Tim Dalgleish, Anne Böckler‐Raettig, Fynn‐Mathis Trautwein, Tania Singer, Philipp Kanske

**Affiliations:** ^1^ Clinical Psychology and Behavioral Neuroscience Technische Universität Dresden Dresden Germany; ^2^ MRC Cognition and Brain Sciences Unit University of Cambridge Cambridge UK; ^3^ Department of Psychology Julius‐Maximilians‐Universität Würzburg Würzburg Germany; ^4^ Department of Psychosomatic Medicine and Psychotherapy, Faculty of Medicine Medical Center—University of Freiburg Freiburg im Breisgau Germany; ^5^ Social Neuroscience Lab Max Planck Society Berlin Germany

**Keywords:** compassion, empathy, fMRI, negative affect, negative thinking processes, stress, theory of mind

## Abstract

Internalizing symptoms such as elevated stress and sustained negative affect can be important warning signs for developing mental disorders. A recent theoretical framework suggests a complex interplay of empathy, theory of mind (ToM), and negative thinking processes as a crucial risk combination for internalizing symptoms. To disentangle these relationships, this study utilizes neural, behavioral, and self‐report data to examine how the interplay between empathy, ToM, and negative thinking processes relates to stress and negative affect. We reanalyzed the baseline data of *N* = 302 healthy participants (57% female, *M*_age_ = 40.52, *SD*_age_ = 9.30) who participated in a large‐scale mental training study, the ReSource project. Empathy and ToM were assessed using a validated fMRI paradigm featuring naturalistic video stimuli and via self‐report. Additional self‐report scales were employed to measure internalizing symptoms (perceived stress, negative affect) and negative thinking processes (rumination and self‐blame). Our results revealed linear associations of self‐reported ToM and empathic distress with stress and negative affect. Also, both lower and higher, compared to average, activation in the anterior insula during empathic processing and in the middle temporal gyrus during ToM performance was significantly associated with internalizing symptoms. These associations were dependent on rumination and self‐blame. Our findings indicate specific risk constellations for internalizing symptoms. Especially people with lower self‐reported ToM and higher empathic distress may be at risk for more internalizing symptoms. Quadratic associations of empathy‐ and ToM‐related brain activation with internalizing symptoms depended on negative thinking processes, suggesting differential effects of cognitive and affective functioning on internalizing symptoms. Using a multi‐method approach, these findings advance current research by shedding light on which complex risk combinations of cognitive and affective functioning are relevant for internalizing symptoms.

## Graphical abstract

We used a multi‐method approach to disentangle risk combinations for internalizing symptoms. Quadratic effects of empathy‐ and theory of mind‐related brain activation on internalizing symptoms depended on negative thinking, highlighting the importance of a nuanced exploration of individual differences and interactions when examining vulnerability to internalizing symptoms.

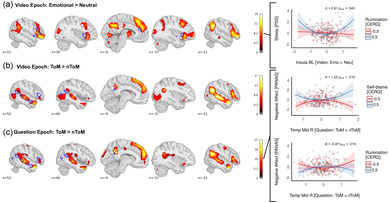

**Practitioner Points**

- The current study aimed to disentangle complex risk combinations of cognitive and affective functioning that are relevant for internalizing symptoms.
- People with lower self‐reported theory of mind (ToM) and higher empathic distress may be at risk for more internalizing symptoms.
- Quadratic effects of empathy‐ and ToM‐related brain activation on internalizing symptoms depended on negative thinking processes and highlighted the importance of investigating nonlinear associations.

## 1 INTRODUCTION

Elevated feelings of stress and negative affect are common mental health problems (Ochnik et al., 2021; Teachman, 2006). These internalizing states are not only prevalent in clinical samples (Hepp et al., 2017; Watson et al., 2011) but also occur in the wider population as part of an emotional spectrum (Rodríguez‐Rodríguez et al., 2022; Teachman, 2006). When feelings of stress and negative affect become more pronounced, intense, or lasting, they are considered key features of mental disorders such as depression and anxiety disorders (Kring, 2008). Moreover, previous research has shown that elevated perceived stress and subthreshold depression increase the risk of depression onset (Cristóbal‐Narváez et al., 2022; Cuijpers, 2004). This raises the question of which specific processes contribute to the development of internalizing symptoms in healthy adults as potential antecedents and early warning signs of mental disorders. Internalizing symptoms encompass a spectrum of mental states relating to disrupted mood and emotions, such as stress, depression, or anxiety (Kovacs & Devlin, 1998). There has been extensive research on different factors that foster internalizing symptoms spanning genetic, psychological, and socio‐environmental factors (Lynch et al., 2021). For instance, Tone and Tully (2014) proposed in a comprehensive theoretical framework that the interplay of maladaptive intermediate states of empathy and theory of mind (ToM) with negative thinking may be linked to internalizing symptoms. As examples of maladaptive intermediate states of empathy and ToM, Tone and Tully (2014) name empathic distress and excessive ToM. Specifically, the authors suggested that the liability to negative thinking processes (e.g., rumination) and hyperarousal play a moderating role in the aberrant development of empathy and ToM, resulting in negative affect or anhedonia. This framework is based on past research showing that not only alterations in empathy and ToM but also negative thinking processes are associated with depression and internalizing symptoms such as negative affect and stress (Förster et al., 2022; Kirkegaard Thomsen, 2006; Nitschke & Bartz, 2023; Schreiter et al., 2013; Zahn et al., 2015). However, previous studies only investigated bivariate associations between these processes or primarily relied on self‐report data (e.g., Powell, 2018; Tully et al., 2016). Considering behavioral data and neural underpinnings of the involved processes may help to disentangle these associations by introducing an additional, nonsubjective perspective. Probing the proposed model of Tone and Tully (2014) using a multi‐method approach with behavioral and fMRI data in addition to self‐reports is essential to unravel the complex relationship between these socio‐affective and ‐cognitive processes. This is why our overarching research questions in this study are: How do empathy and ToM relate to internalizing symptoms not only on a self‐report level but also on the behavioral and neural level? And is their association moderated by negative thinking processes?

Empathy and ToM are both multifaceted constructs and different definitions in the research literature exist (Decety & Jackson, 2004; Frith & Frith, 2005; Kanske, 2018; Premack & Woodruff, 1978; Shamay‐Tsoory, 2011; Singer, 2006). Here, we understand empathy as an affective process of sharing others' emotions (de Vignemont & Singer, 2006). ToM, on the other hand, also often referred to as perspective taking, mentalizing, or cognitive empathy, can be defined as inferring other people's mental states (Frith & Frith, 2005). As such, both processes are distinct but can interact (Kanske et al., 2016; Maliske et al., 2023). Yielding access to others' inner mental states, empathy and ToM are crucial for daily social interactions (Singer, 2012). But in situations that involve sharing or reasoning about other people's suffering, they may lead to undesired emotional states such as interpersonal guilt or empathic distress (Klimecki & Singer, 2011). For instance, seeing that a friend is very sad can evoke either feelings of healthy empathic sadness or of empathic distress in the observer. Also, overthinking a friend's mental state (high ToM) could lead to being overly sensitive to their expectations and thus feeling guilty. On the other hand, difficulties in taking their perspective (low ToM) could lead to problems in reading social cues correctly and thus to secondary stress in these social situations. In order to change these negative emotions into a more desired or positive emotional state, people use different emotion regulation strategies (Förster et al., 2022; Tull & Aldao, 2015). However, some emotion regulation strategies can also be classified as dysfunctional negative thinking processes (i.e., *rumination* or *self‐blaming thoughts*; Loch et al., 2011). Both rumination and self‐blame are cognitive strategies that may initially be used to deal with aversive emotional states, but as such, they can fail to reduce negative emotions and even exaggerate the already negative and self‐oriented emotional state (Mor & Winquist, 2002; Powell, 2018).

Since Tone and Tully (2014) introduced their framework, only a few studies evaluated the suggested associations (Gray & Tully, 2020; Powell, 2018; Tully et al., 2016). These investigations mainly probed quadratic relationships, for instance, whether both low and high (compared to moderate) levels of empathy and ToM are associated with internalizing symptoms (Powell, 2018). Yet, the studies yielded diverging results: Whereas Tully et al. (2016) reported that higher and lower ToM was associated with depressive symptoms, Powell (2018) did not find this quadratic pattern. These inconclusive results indicate that the interplay of empathy, ToM, negative thinking, and affect is complex. Especially potential quadratic associations need further exploration, for example, by complementing self‐report with more objective measures, such as neural data.

Reviewing research on neural correlates of empathy, ToM, emotion regulation, stress, and negative affect reveals an overlap of related brain networks (Berretz et al., 2021; Förster et al., 2022; Lindquist et al., 2016). The activation in the anterior insulae (AI), inferior frontal gyri (IFG), amygdalae, and the left anterior cingulate cortex (ACC) seems not only to be associated with empathy (Bzdok et al., 2012; Lamm et al., 2011; Schurz et al., 2021; Singer et al., 2004; Stietz et al., 2019) but also with negative affect (Lindquist et al., 2016) and psychological stress (Berretz et al., 2021). Overlaps between the ToM network and neural correlates of stress, negative affect, or rumination encompass the dorsomedial prefrontal cortex and middle temporal gyrus, but also precuneus (Berretz et al., 2021; Bzdok et al., 2012; Jacob et al., 2020; Lindquist et al., 2016; Schurz et al., 2021). These processes recruit a multifunctional network of brain areas that dynamically interact and could indicate a functional association between empathy, ToM, negative thinking processes, and internalizing symptoms. However, few studies examined these associations at the level of the brain while considering the interactive relationships, for example, between internalizing symptoms and empathy or ToM (Guendelmann et al., 2022).

Consequently, we aim to investigate how the interplay of empathy, ToM, and negative thinking affects internalizing symptoms in a healthy sample by considering multiple methods. Based on the literature, we first formulate two clear expectations. Additionally, we pose two exploratory research questions, as previous research does not allow explicit assumptions about how neural correlates of empathy and ToM specifically relate to both negative thinking and internalizing symptoms.

In summary, we know little about whether the association of experimentally assessed empathy and ToM abilities with internalizing symptoms on a subclinical level is moderated by negative thinking processes. However, based on self‐report studies, we assume that negative thinking processes, such as rumination and self‐blame, moderate the effects of self‐report and behaviorally assessed empathy and ToM on stress and negative affect (research question 1).

Second, previous investigations point toward nonlinear relationships of empathy, ToM, negative thinking, and internalizing symptoms but yielded inconclusive results (Powell, 2018; Tully et al., 2016). Hence, we expect that the regression models testing our first expectation can be improved by adding quadratic terms of empathy and ToM measures (research question 2).

Third, as past studies did not use fMRI data to investigate interactive relationships of empathy and ToM with internalizing symptoms, we explore whether there are different neural patterns of empathy and ToM on a whole brain level depending on individual stress and negative affect levels (research question 4).

Finally, it remains unclear whether empathy‐ and ToM‐related neural activation is associated with internalizing symptoms in interaction with negative thinking processes. Thus, we explore whether rumination and self‐blame moderate the association of empathy‐ and ToM‐related brain activity and internalizing symptoms (research question 4a) and whether these associations are nonlinear (research question 4b).

## 2 METHODS

### 2.1 Sample

For the present investigation, we performed a secondary data analysis to the baseline data (T0) of a large longitudinal randomized controlled trial, a 9‐month longitudinal mental training study, the ReSource study (Singer et al., 2016). The study was registered at ClinicalTrial.org (“Plasticity of the Compassionate Brain”, Identifier NCT01833104) and approved by two local ethics committees (Leipzig: 376/12‐ff; Berlin: 2013–20, 2013–29, 2014–10). Between 2012 and 2014, participants were recruited at two sites in Germany. Recruitment followed a multistep procedure, including screening, interviews, and questionnaires. For a detailed description of the sample, recruitment strategies, and the complete list of exclusion criteria, see Singer et al. (2016). Exclusion criteria relevant for the present investigation encompassed the following (see Singer et al., 2016, p. 48f.): Age <20 and >55; not speaking/understanding German fluently; not meeting MRI safety standards (e.g., neurological disorders, pregnancy, tattoos on the upper part of the body); psychotherapy within the last 2 years, diagnosed mental disorders (unless symptom‐free >2 years); medications (e.g., psychotropics, opiates, corticosteroids, or for psychological problems); drugs and alcohol abuse; smoking >5 cigarettes per week; current mild to severe depression symptoms (Major Depression Inventory; Bech et al., 2001); high trait anxiety levels (State–Trait Anxiety Inventory >56; Laux et al., 1981); and high alexithymia (Toronto Alexithymia Scale‐20 > 60; Bagby et al., 1994; Kupfer et al., 2001). Participants were reimbursed for the measurement time and gave written informed consent before the study.

**FIGURE 1:**
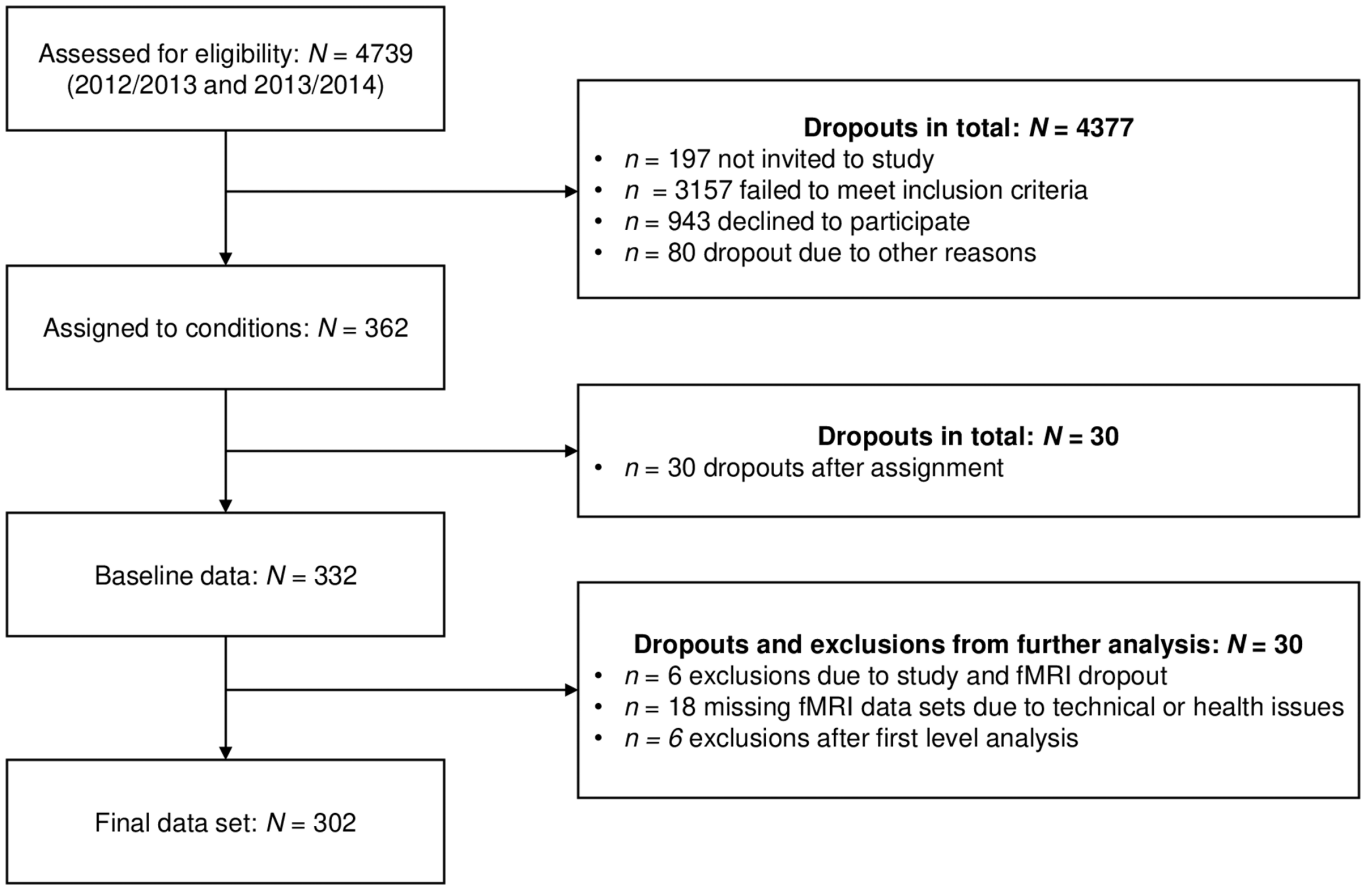
Flow diagram of study participants.

Figure 1 displays a participant flow diagram. In total, *N* = 332 participants completed baseline measurements. However, for the present investigation, *n =* 30 participants had to be excluded from the analyses (*n* = 18 missing datasets due to technical or health issues, *n* = 6 dropouts of study or MRI, *n* = 6 exclusions after the first level analysis because of problems with the degree of freedom estimation). Due to partially missing questionnaire data (see Table 1), the final sample size varies slightly between the respective analyses.

**TABLE 1:** Sample characteristics of the reanalyzed ReSource dataset.

Demographic variables^a^	*n* (%)	Missing (*n*)
*Gender*		
Female	173 (57%)	
Male	129 (43%)	
Education		27
Other educational training	100 (36%)	
PhD	21 (7.6%)	
University degree	154 (56%)	
Income (€)		27
<1000	40 (15%)	
1000 to <2000	74 (27%)	
2000 to <4000	97 (36%)	
4000 to <5000	32 (12%)	
≥5000	32 (12%)	

	Mean (SD)	Range	Missing (*n*)
Age	40.52 (9.30)	20.00–55.00	
IQ	115.14 (15.12)	78.00–157.00	5
PSS‐10 stress	14.21 (5.75)	2.00–35.00	6
PANAS negative affect	9.01 (5.14)	0.00–31.00	5
CERQ self‐blame	1.30 (0.74)	0.00–3.67	4
CERQ rumination	1.79 (0.85)	0.00–4.00	4
IRI empathic concern	2.63 (0.52)	1.00–3.86	2
IRI personal distress	1.66 (0.57)	0.14–3.29	2
IRI perspective taking	2.51 (0.54)	1.14–4.00	2
EmpaToM compassion mean	3.41 (0.75)	0.73–5.42	
EmpaToM valence contrast	1.52 (0.75)	−0.70–3.29	
EmpaToM ToM performance	0.00 (0.39)	−1.20–1.09	

*Note*: Sample description may vary from other ReSource publications due to missing variables relevant to the present investigation.

Abbreviations: CERQ, Cognitive Emotion Regulation Questionnaire; IRI, Interpersonal Reactivity Index; PANAS, Positive Affect Negative Affect Scale; PSS‐10, Perceived Stress Scale.

^a^ *N* = 302 participants are included in the descriptive analysis.

### 2.2 Procedure and measures

Participants completed several questionnaires, behavioral tasks, and physiological assessments at baseline. Many of the present measures and data have already been published in different contexts in previous ReSource project publications (Böckler et al., 2017, 2018; Engert et al., 2017, 2018; Hildebrandt et al., 2019; Kanske et al., 2015; Liebmann et al., 2023; Puhlmann et al., 2021; Tholen et al., 2020; Trautwein et al., 2020; Valk et al., 2016, 2017). It should be noted that the analyses of the study presented here and previous studies using the same measures are based on slightly different samples. This mainly concerns data from the EmpaToM task, measuring empathy and ToM, as early publications on the initial validation of this novel paradigm could not yet include all participant cohorts of this large‐scale longitudinal study (Kanske et al., 2015). Missingness depends on the respective combination of measures and analyses in a given paper (for more details, see Table S1 in the supplementary materials; Kanske et al., 2015; Tholen et al., 2020; Trautwein et al., 2020). However, none of these previous papers investigated the concrete relationship between empathy, ToM with negative thinking processes, and internalizing symptoms using the baseline dataset (T0) of the ReSource project combined. Differences from previous studies are highlighted. Specifically, the (quadratic) regression analyses, including brain activation of a priori defined regions of interest (ROIs), and the whole brain analyses, including interactions with covariates, have not been conducted before. For the present investigation, the following self‐report and behavioral measures are relevant.

#### 2.2.1 Questionnaires

Demographic data included age, gender, income, family status, and education.

The Perceived Stress Scale (PSS‐10; Cohen et al., 1983) assesses self‐perceived stress across the last month with 10 items. The items are rated on a 5‐point response scale ranging from 0 (*never*) to 4 (*very often*). We used a sum score, whereas higher values indicate more stress. Cronbach's alpha in our sample was *α* = .87, showing good internal consistency. The perceived stress scale showed a significant positive Spearman's correlation with the depression sum score (calculated with the Beck Depression Scale II [BDI II]; Beck et al., 1996): *r* = .53, 95% CI [0.44, 0.61], *p* < .001.

*The Positive and Negative Affect Schedule* (PANAS; Krohne et al., 1996) measures positive and negative affect with 20 items. Participants were asked to rate 10 negative and 10 positive emotions with respect to how they felt during the last weeks (e.g., guilt, shame) on a 5‐point response scale ranging from 0 (*not at all*) to 4 (*a lot*). A sum score of the *negative affect* subscale (Cronbach's *α* = .84) was used to measure negative affect. The negative affect subscale showed a significant positive Spearman's correlation with the depression sum score (calculated with the BDI II; Beck et al., 1996): *r* = .49, 95% CI [0.40, 0.57], *p* < .001.

The *Cognitive Emotion Regulation Questionnaire* (CERQ; Loch et al., 2011) is a questionnaire that assesses nine different emotion regulation strategies with 36 items overall. We used the two subscales *self‐blame* and *rumination* to measure negative thinking processes, such as the tendency to blame oneself for mistakes and difficulties to stop thinking about past situations. For each of these subscales, four items are rated on a 5‐point response scale ranging from 1 (*almost never*) to 5 (*almost always*). For the German version, a sparser version with three items per scale was recommended that provides equally good reliability and validity; that is why we used a three‐item solution to calculate the mean of the subscales (for details, see Loch et al., 2011). Higher values indicate more self‐blaming thoughts and rumination. In our sample, the internal consistency (Cronbach's alpha) for the rumination subscale was *α* = .65, and for the self‐blame subscale, it was *α* = .68.

The *Interpersonal Reactivity Index* (IRI; Davis, 1983) includes 28 items and assesses different aspects of empathy using four subscales with seven items each. Items are rated on a 5‐point response scale ranging from 0 (*does not describe me well*) to 4 (*describes me very well*). Here, we used three subscales: First, the *personal distress* subscale (Cronbach's *α* = .76) captures feelings of discomfort when facing negative experiences of others. Second, the *empathic concern* subscale (Cronbach's *α* = .72) measures compassionate feelings toward others, and last, the *perspective taking* subscale (Cronbach's *α* = .77) assesses cognitive reasoning of other people's mental states. Higher values indicate more personal (empathic) distress, compassion, and perspective taking.

#### 2.2.2 EmpaToM task

**FIGURE 2:**
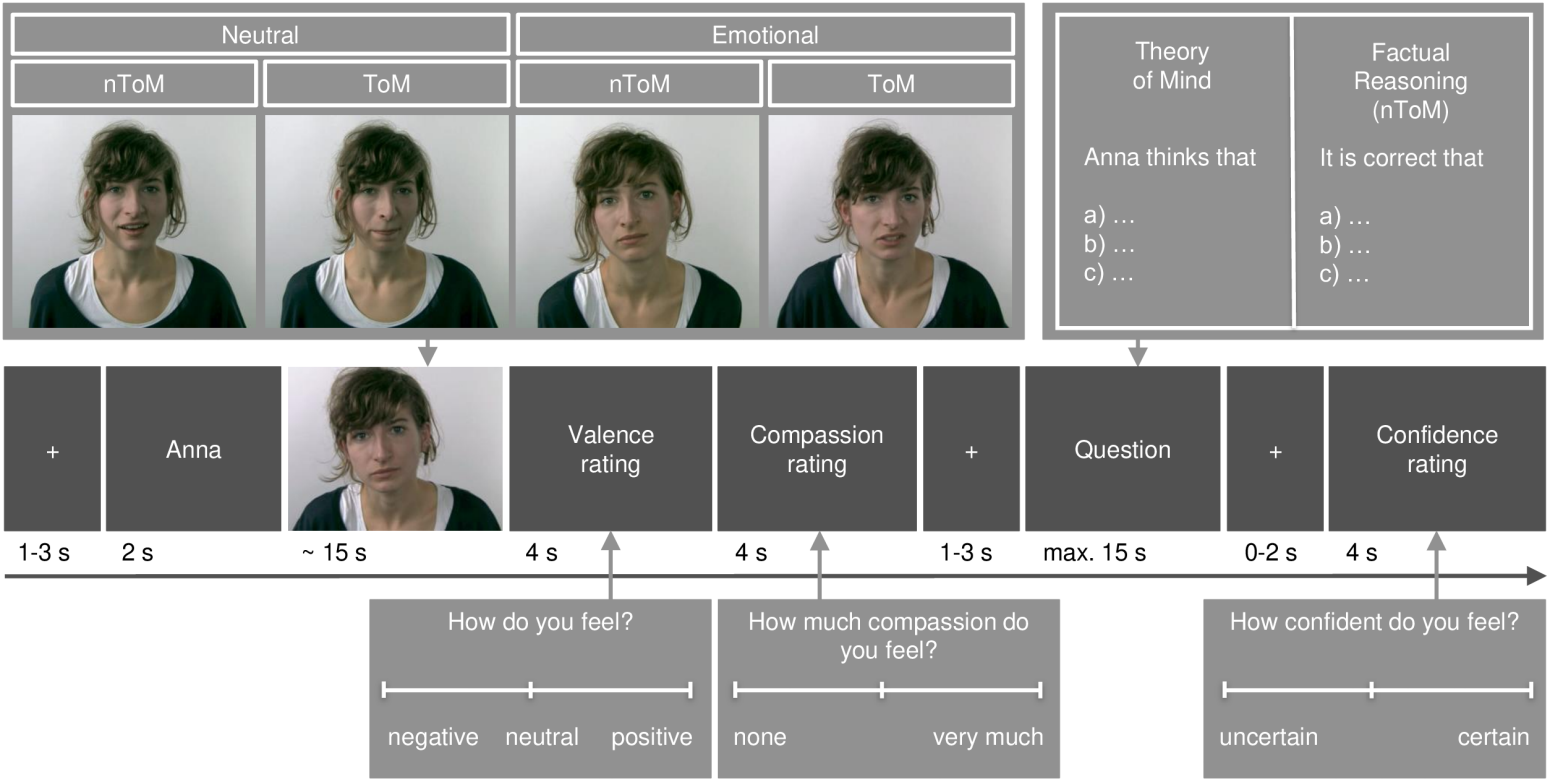
An exemplary trial sequence of the EmpaToM task; the figure is adapted from Kanske et al. (2015).

The *EmpaToM* task is an experimental fMRI paradigm measuring empathy and ToM using naturalistic video material (Kanske et al., 2015; Tholen et al., 2020). In detail, the EmpaToM follows a 2 × 2 factorial design including four conditions: The valence factor consists of the conditions *neutral* and *emotional* videos. The ToM factor consists of the conditions *factual reasoning* and *ToM* videos and questions. Please note that we will use the abbreviation nToM for the factual reasoning condition. In total, the task contains 48 trials with 12 trials for each condition. Thus, the number of trials is equal across conditions. Figure 2 displays one exemplary trial sequence: After a fixation cross (1–3 s), the actor's name is displayed (2 s), followed by a video in which a narrator either talks about emotional or neutral personal experiences (*video epoch*: max. 15 s). The content also varies in terms of ToM demand (ToM vs. nToM). Ratings of valence (*negative* to *positive*) and compassion (*none* to *very much*) follow (each 4 s). After a second fixation cross (1–3 s), participants complete a multiple‐choice question (*question epoch*: max. 14 s) that requires either ToM skills (e.g., reasoning about the actor's goals) or factual reasoning (nToM). Finally, after a fixation cross (0–2 sec), confidence ratings (4 s) capture the certainty of the participant (*uncertain* to *certain*) regarding their previous multiple‐choice performance.

We analyzed empathy, compassion, and ToM performance measures in the same way as already reported by Kanske et al. (2015). Behavioral empathy is operationalized as the emotional reactivity captured by the reversed valence contrast (emotional > neutral videos). Higher empathy indicates more emotion sharing when witnessing emotionally negative compared to neutral content. Compassion is operationalized as the mean of compassion ratings across emotional and neutral conditions. Higher values indicate more empathic concern and warm feelings toward the narrators in the videos, irrespective of the emotional content. To measure ToM, we calculated a mean score of reversed reaction times and accuracy scores of the ToM demanding questions.

We used the contrast of emotional versus neutral video epochs to measure empathy‐related brain activation. To measure ToM‐related brain activation, we considered two trial epochs: By extracting brain activation during the video epoch (ToM vs. nToM trials), a more implicit measure of ToM‐processing can be derived. Extracting contrast images (ToM vs. nToM trials) during the question epoch yields an explicit measure of ToM.

### 2.3 fMRI acquisition

A 3 Tesla MRI scanner (Siemens Magnetom Verio Siemens Medical Solutions, Erlangen, Germany) with a 32‐channel head coil was used for fMRI acquisition. For functional images, a T2*‐weighted echo‐planar imaging sequence was applied (TR = 2000 ms; TE = 27 ms, flip angle of 90°, matrix = 70 × 70 mm, FOV = 210 mm). The whole brain was covered by acquiring 37 axial slices of 3 mm per TR. Structural images were acquired using an MPRAGE T1‐weighted sequence (TR = 2300 ms; TE = 2.98 ms; TI = 900; flip angle = 9°; 176 sagittal slices; matrix size = 256 × 256; FOV = 256 mm; slice thickness = 1 mm), yielding a final voxel size of 1 × 1 × 1 mm. The first three volumes of each run were discarded from the analyses.

### 2.4 Data analysis

#### 2.4.1 Preliminary analysis

All analyses of questionnaires and behavioral data were done using R version 4.0.3 (R Core Team, 2020). First, as some of the variables were not normally distributed, multiple Spearman's correlations were conducted to describe bivariate associations of questionnaire data (self‐perceived stress, rumination, self‐blame, IRI personal distress, IRI empathic concern, IRI perspective taking) with behavioral measures of empathy and ToM (measured with the EmpaToM) and brain activation associated with empathy and ToM in a priori defined ROIs. We did not adjust for multiple testing for these exploratory, descriptive analyses.

The EmpaToM valence ratings, compassion ratings, and ToM performance scores were analyzed with two‐way repeated measures ANOVAs, including the two within‐subject factors valence (emotional vs. neutral content) and ToM requirement (ToM vs. nToM). Even though results of the EmpaToM behavioral data have been reported previously (Kanske et al., 2015; Tholen et al., 2020; Trautwein et al., 2020), we still conducted an ANOVA for the following reasons: First, in contrast to the reported results from Tholen et al. (2020), we combined the EmpaToM data in one sample, and results may differ for this reason. Second, although Trautwein et al. (2020) also used the combined sample and behavioral EmpaToM data, no ANOVA was conducted for the baseline data, as they focused on reporting the training‐related longitudinal changes in EmpaToM measures to test for differential effects of different mental training modules.

#### 2.4.2 Research question 1: Linear associations

We conducted multiple linear regression analyses to follow up on the findings of Tully et al. (2016) and Powell (2018) and to investigate our first research question, whether negative thinking processes moderate the effects of empathy and ToM on internalizing symptoms. In multiple regression models, we probed the association of the independent variables (IVs) IRI personal distress, IRI empathic concern, and IRI perspective taking with the two outcome variables stress and negative affect. For each IV, we computed one model to describe stress (outcome 1) and one to describe negative affect (outcome 2). Each model contained in total seven terms: Main effects of two covariates (age and gender), main effects for three effects of interest (self‐blame, rumination, and the respective IV, e.g., personal distress), and two interaction terms (IV × self‐blame and IV × rumination). We repeated this procedure with the task‐based EmpaToM valence contrast, compassion mean, and ToM performance score (calculated according to previous studies; Kanske et al., 2015). Numeric IVs were scaled by two times the standard deviation of the respective variable. Outcome variables were scaled by one standard deviation before entering the models. This procedure allows direct comparison of regression coefficients of continuous and (untransformed) binary predictors (Gelman, 2008).

#### 2.4.3 Research question 2: Quadratic associations

As we also wanted to probe whether potential associations are nonlinear (research question 2), we added the quadratic terms of IRI personal distress, IRI empathic concern, IRI perspective taking, EmpaToM valence contrast and ToM performance score, as well as EmpaToM compassion mean with self‐blame and rumination in a second step to the respective models. Thus, in each model next to the two interaction terms from the first step (e.g., stress = self‐blame × personal distress + rumination × personal distress + age + gender), the two nonlinear interaction terms were added to the other IVs in a second step (e.g., self‐blame × [personal distress]^2^ + rumination × [personal distress]^2^). We compared the model performance of the linear versus the quadratic models with the *check_performance*() function (Lüdecke et al., 2021). This function compares multiple fit indices, including *R* ^2^, corrected *R* ^2^, Bayesian information criterion, Akaike information criterion, root mean squared error, and residual standard deviation (Sigma), and ranks the models based on their overall performance. Model comparisons are included in the supplements (see Tables S5–S10).

#### 2.4.4 Additional analyses

Assumptions of the linear and nonlinear regression models were checked with the *check_model*() function (Lüdecke et al., 2021). In case of outliers or deviations from normality, we calculated bootstrapped estimates with 1000 iterations. We only report bootstrapped estimates if the model outcome has changed. If not otherwise indicated, the original regression models are reported. To adjust for multiple testing, we adjusted *p*‐values (*p*_adj._) in each model using the false discovery rate (FDR) and, thus, taking the number of IVs per model into account (Benjamini & Hochberg, 1995).

In addition, because the main effects from models that include interaction terms are difficult to interpret, we removed the interaction terms from the models in the case of nonsignificant interactions, as suggested by Lorah (2020). This allowed us to examine empathy‐ and ToM‐related main effects in more detail.

We finally used a sensitivity analysis to determine the minimum detectable effect size (for details, see supplements). For linear multiple regression (fixed model) with *α* = .05 (two‐tailed), *β* = .2 (assumed power = .8), and given the sample size *N* = 296, the sensitivity analysis for the linear models, including seven predictors, yielded a critical *t* = 1.97 (df = 288) and a minimum detectable effect size of *f* ^2^ = 0.027 (*d* = 0.32). The sensitivity analyses for the quadratic models, including 10 predictors, resulted in a critical *t* = 1.97 (df = 285) and a minimum detectable effect size of *f* ^2^ = 0.027 (*d* = 0.32). Thus, we were able to detect small effects with our regression models.

#### 2.4.5 fMRI data analysis

##### fMRI preprocessing

Preprocessing was identical to previous reports and is described in detail there (using SPM8; Kanske et al., 2015; Tholen et al., 2020). Briefly, preprocessing steps included coregistration, slice‐time correction, and realignment. Also, images were normalized (3 mm isotropic volumes), spatially smoothed (full‐width half maximum: 8 mm), and a high‐pass temporal filter was applied (128 s).

##### First level analysis

For the first and second level analyses of the current study, SPM12 (https://www.fil.ion.ucl.ac.uk/spm/software/spm12/) was used within a MATLAB 9.11 environment (MathWorks Inc., Sherborn, MA). On the first level, we used the RWLS toolbox (Diedrichsen & Shadmehr, 2005) that implements a restricted maximum likelihood approach during model specification and estimation. In detail, variance is estimated to weigh down observations with higher noise to yield more robust estimates.

As neural correlates of ToM can be derived from both the video and question epoch, we specified a general linear model and included regressors indicating onset times for the four conditions (neutral, emotional, nToM, ToM) of both video and question epochs. In sum, the general linear model contained these eight regressors (four per video and four per question epoch) and regressors for the empathy, compassion, and confidence ratings. Also, we controlled for head movement by adding six movement parameters to the design matrix as regressors of no interest. Regressors were convolved with a canonical hemodynamic response function.

After model estimation, we added contrast weights to the respective estimates and defined the contrasts of interest: empathy contrast during the video epoch (emotional > neutral), the ToM contrast during the video epoch (ToM > nToM) as well as the ToM contrast during question epoch (ToM > nToM).

##### Research question 3: Effects on the whole brain level

First, we used simple one‐sample *t* tests of the empathy and the two ToM contrasts (during video and question epochs) to capture empathy‐ and ToM‐related brain activation. These analyses are the same as already reported by Kanske et al. (2015) and Tholen et al. (2020), but with the total baseline sample of the ReSource project (*N* = 302; see Supplementary Table S1 for details about differences in sample sizes and missingness).

Second, we used whole brain analyses to answer our third research question, whether empathy and ToM‐related brain activation differs depending on individual stress and negative affect levels (research question 3). This analysis has not been conducted before. In detail, we specified and estimated a flexible factorial model with three factors: (a) subject (to account for subject variance), (b) empathy (neutral vs. emotional), and (c) ToM (nToM vs. ToM) and the respective covariates (negative affect and stress). The contrast of interest was the interaction between the respective covariate and the emotionality or ToM factor (e.g., stress × empathy or stress × ToM). After model specification and estimation, we added linear contrast weights to the respective estimates. Brain activation that passes correction during these contrasts indicates that depending on the covariate (e.g., low vs. high perceived stress), brain activation for the respective factor (emotionality, implicit ToM, and explicit ToM) varies. For instance, people with higher perceived stress show higher activation in specific areas during emotional compared to neutral videos. As we used flexible factorial models and not simple *t* tests, all main and interaction effects were controlled for the respective other factors (including the subject factor).

##### Research question 4: Effects of ROIs

To investigate our final exploratory research question, whether negative thinking processes moderate the effects of empathy‐ and ToM‐related brain activity on internalizing symptoms, we extracted brain activation of predefined ROIs and used the respective estimates in linear and nonlinear regression analyses. To extract activation, we used openly available *T* maps (https://identifiers.org/neurovault.collection:821) derived from a previous meta‐analysis (Bzdok et al., 2012). As the statistical maps of Bzdok et al. (2012) covered rather large areas of the brain, we decided to restrict the respective clusters to areas that are not only associated with empathy or ToM but also with both negative affect and stress (for meta‐analyses, see Berretz et al., 2021; Lindquist et al., 2016). Therefore, we first extracted binary masks of the structural regions that were previously associated with stress and negative affect (for meta‐analyses, see Berretz et al., 2021; Lindquist et al., 2016) by using the automatic anatomical labeling (AAL) WPU PickAtlas (see Supplementary Table S3 for an overview of all ROIs). Second, only for those regions that were associated with both stress or negative affect and empathy or ToM, we used the minimum statistics of SPM's imcalc to extract the conjunction between these regions and the empathy‐ or ToM‐related brain masks of Bzdok et al. (2012). This procedure resulted in five empathy‐related ROIs: Bilateral AI, right IFG (pars triangularis), right amygdala, left ACC, and left medial prefrontal cortex (mPFC). Because previous reports have not consistently linked ACC and mPFC activation to stress (Berretz et al., 2021), we used the ACC and the mPFC ROIs only to test their association with negative affect, not stress. Additionally, we employed the same procedure using the conjunction of the ToM‐related meta‐analytical *T* map (Bzdok et al., 2012) and anatomical regions associated with stress and negative affect (Berretz et al., 2021; Lindquist et al., 2016). This resulted in four ToM‐related ROIs, including the right IFG (pars triangularis), right middle temporal gyrus, left precuneus, and left mPFC. Please note that we used the precuneus ROI only to describe stress but not negative affect, as there was no meta‐analytic evidence for an association between precuneus activation and affect (Lindquist et al., 2016). Conversely, we used the mPFC ROI only to describe negative affect (for associations, see Lindquist et al., 2016), but not stress.

The resulting empathy‐related masks were then used in Marsbar to extract mean brain activation based on the one sample *t* test for the valence contrast emotional > neutral (video epoch). Since we did not find significant brain activation in the right amygdala ROI, we did not use this ROI for further analysis (see Supplementary Table S4). The ToM‐related masks were employed to extract activation based on the contrasts ToM > nToM (video epoch) and ToM > nToM (question epoch). Finally, we repeated the procedure described for the behavioral and questionnaire data to investigate research questions 1 and 2. Thus, in the first step, we calculated regression models for each ROI separately to describe stress and negative affect in interaction with self‐blame and rumination (research question 4a). In the second step, we introduced quadratic terms of the ROI estimates to each model (research question 4b).

## 3 RESULTS

### 3.1 Participants and descriptive analysis

**FIGURE 3:**
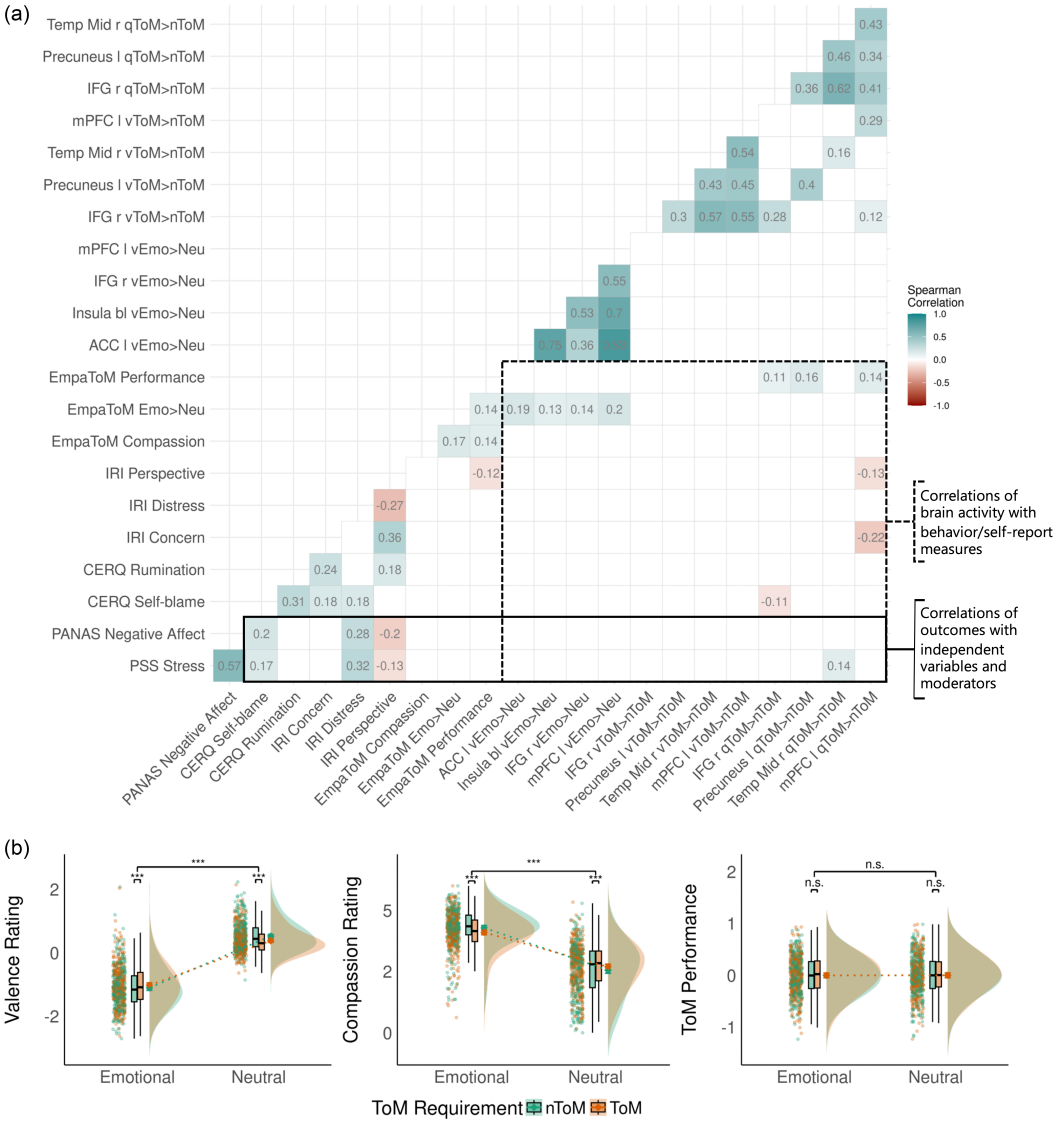
Panel (a) shows a heatmap with significant pairwise Spearman's correlation coefficients of all measures of interest. Panel (b) displays the results of the EmpaToM valence, compassion ratings, and ToM performance in raincloud plots analyzed with three 2‐way repeated measures analyses of variance.

Table 1 provides a detailed description of the sample and the mean values of relevant measures. Pairwise correlation coefficients of all measures of interest are displayed in Figure 3a.

Of the *n* = 302 participants, *n* = 153 (51%) were recruited in Leipzig. Participants were on average *M* = 40.52 years old (*SD* = 9.30; range = 20–55 years), and *n* = 173 (57%) participants were female. Please note that the descriptive sample characteristics differ slightly from previous reports (Kanske et al., 2015; Tholen et al., 2020; Trautwein et al., 2020) due to the exclusion of incomplete cases relevant to the current combination of measures and respective analyses.

The behavioral EmpaToM measures were analyzed following Kanske et al. (2015), but now with the baseline data of all cohorts of the ReSource project combined. Regarding the valence ratings, there were significant main effects for the emotional content of videos, *F*(1, 301) = 1249.45, *p <* .001, *η*_p_^2^ = 0.81, and ToM requirement, *F*(1, 301) = 6.44, *p =* .012, *η*_p_^2^ = 0.02. A significant interaction effect, *F*(1, 301) = 71.00, *p <* .001, *η*_p_^2^ = 0.19, indicated more negative affect after nToM compared to ToM demanding videos in emotional trials, *t*(301) = −5.39, *p*_adj._ < .001. In neutral trials, affect ratings were more positive after nToM than ToM demanding videos, *t*(301) = 6.83, *p*_adj._ < .001.

Compassion ratings were higher for emotional than neutral videos, indicated by a significant main effect, *F*(1, 301) = 865.59, *p <* .001, *η*_p_^2^ = 0.74. The main effect of the ToM requirement was not significant, indicating no significant differences in compassion ratings after ToM compared to nToM demanding videos, *F*(1, 301) = 1.92, *p* = .167, *η*_p_^2^ = 0.01. A significant interaction effect, *F*(1, 301) = 93.19, *p <* .001, *η*_p_^2^ = 0.24, suggested more compassion after nToM compared to ToM demanding videos in emotional trials, *t*(301) = −5.19, *p*_adj._ < .001, whereas in neutral trials, compassion ratings were lower for nToM compared to ToM demanding videos, *t*(301) = 9.05, *p*_adj._ < .001.

ToM performance scores did not significantly differ between emotional or neutral trials, *F*(1, 301) = 0.04, *p* = .843, *η*_p_^2^ = 0.00, or Tom versus nToM demanding videos, *F*(1, 301) = 0.00, *p* = .949, *η*_p_^2^ = 0.00. There was no significant interaction, *F*(1, 301) = 0.00, *p* = .968, *η*_p_^2^ = 0.00.

### 3.2 Research question 1: Linear associations

We calculated several multiple linear regressions to investigate the potential moderating effects of negative thinking processes (self‐blame and rumination) on the association of self‐reported and behavioral markers of empathy, compassion, and ToM with internalizing symptoms (research question 1).

#### 3.2.1 Empathy

We aimed to describe stress during the last month (PSS‐10) and negative affect during the previous weeks (PANAS negative affect subscale) by the interaction of negative thinking processes with self‐report as well as behavioral measures of empathy (IRI personal distress vs. EmpaToM valence contrast). This resulted in four models (see Table 2). The models revealed no significant interaction effects. However, there were main effects of IRI personal distress on stress, *ß* = .57, 95% CI [0.34, 0.79], *p*_adj._ = < .001, and negative affect, *ß* = .50, 95% CI [0.28, 0.73], *p*_adj._ < .001, indicating that higher levels of personal (empathic) distress were associated with higher levels of internalizing symptoms. Also, self‐blame was significantly related to negative affect when including the IRI personal distress subscale (for estimates, see Table 2). The main effect of the valence contrast (valence ratings after emotional > neutral videos) on stress, suggesting that stronger emotional reactivity toward emotional stimuli is associated with stress, did not pass FDR correction, *ß* = .25, 95% CI [0.03, 0.48], *p*_adj._ = .103.

**TABLE 2:** Regression models, including self‐report and behavioral measures of empathy to predict stress and negative affect.

	Outcome 1: Stress						Outcome 2: Negative affect					
	Self‐report empathy measure (IRI) as IV			Behavioral empathy measure (EmpaToM) as IV			Self‐report empathy measure (IRI) as IV			Behavioral empathy measure (EmpaToM) as IV		
Predictors	*ß*	95% CI	*p*_adj._	*ß*	95% CI	*p*_adj._	*ß*	95% CI	*p*_adj._	*ß*	95% CI	*p*_adj._
(Intercept)	−.05	−0.19 to 0.10	.758	−.00	−0.15 to 0.14	.949	−.11	−0.26 to 0.03	.202	−.06	−0.21 to 0.09	.667
Age	−.10	−0.32 to 0.12	.738	−.12	−0.35 to 0.11	.479	−.33	−0.55 to −0.11	**.012**	−.38	−0.61 to −0.16	**.008**
Gender [male]	.11	−0.12 to 0.34	.738	.01	−0.22 to 0.24	.949	.21	−0.02 to 0.43	.143	.10	−0.13 to 0.33	.667
Personal distress	**.57**	**0.34 to 0.79**	**<.001**				**.50**	**0.28 to 0.73**	**<.001**			
Rumination	.02	−0.22 to 0.25	.889	.02	−0.22 to 0.26	.949	.13	−0.10 to 0.36	.374	.12	−0.12 to 0.36	.667
Self‐blame	.20	−0.03 to 0.43	.357	**.28**	**0.04 to 0.51**	.103	**.24**	**0.01 to 0.47**	.101	**.33**	**0.10 to 0.57**	**.023**
Personal distress × rumination	.13	−0.33 to 0.59	.758				−.03	−0.48 to 0.43	.903			
Personal distress × self‐blame	.04	−0.40 to 0.47	.889				.16	−0.27 to 0.58	.528			
Valence emo > neu				**.25**	**0.03 to 0.48**	.103				−.05	−0.27 to 0.18	.679
Valence emo > neu × rumination				.44	−0.04 to 0.92	.197				.11	−0.37 to 0.59	.679
Valence emo > neu × self‐blame				.28	−0.19 to 0.76	.477				.15	−0.32 to 0.62	.679
Observations	296			296			296			296		
*R* ^2^/*R* ^2^ adjusted	.110/.088			.067/.045			.146/.125			.086/.064		

Abbreviations: IRI, Interpersonal Reactivity Index; IV, independent variable.

#### 3.2.2 Theory of mind

We explained stress and negative affect by using the interaction of negative thinking processes with self‐report versus behavioral measures of ToM (IRI perspective taking vs. EmpaToM ToM performance score), resulting in the following models: Stress was described by interactions of negative thinking with (a) IRI perspective taking or (b) EmpaToM ToM performance and negative affect was described by interactions of negative thinking with (c) IRI perspective taking or (d) EmpaToM ToM performance. All models were adjusted for age and gender.

IVs only explained a small proportion of variance of stress and negative affect (range *R* ^2^_adjusted_ = .02–.10). IRI perspective taking, but not the EmpaToM ToM performance score, described both stress (model a), *ß* = −.33, 95% CI [−0.56, −0.10], *p*_adj._ = .026, and negative affect (model c), *ß* = −.41, 95% CI [−0.64, −0.18], *p*_adj._ = .004. In both models, lower self‐reported perspective taking was associated with higher levels of internalizing symptoms.

Moreover, models a, c, and d revealed that higher levels of self‐blaming thoughts were associated with higher levels of stress and negative affect while accounting for the respective ToM measures: (a) *ß* = .33, 95% CI [0.09, 0.57], *p*_adj._ = .026, (c) *ß* = .36, 95% CI [0.12, 0.59], *p*_adj._ = .007, (d) *ß* = .34, 95% CI [0.10, 0.58], *p*_adj._ = .019. The interaction of IRI perspective taking and rumination on stress did not pass FDR correction, *ß* = −.53, 95% CI [−1.03, −0.03], *p*_adj._ = .099. Also, none of the interaction terms of self‐blame with both IRI and EmpaToM measures of ToM, nor the interaction of rumination with the ToM performance score were significant (all *p*_adj._ > .05).

#### 3.2.3 Compassion

We also aimed to investigate the relationships of compassion measures with negative thinking processes and internalizing symptoms. Four models were calculated: Stress was described by interactions of negative thinking with (a) IRI empathic concern or (b) EmpaToM compassion and negative affect by interactions of negative thinking with (c) IRI empathic concern or (d) EmpaToM compassion measure. All models were controlled for age and gender. IVs only explained a small proportion of variance of stress and negative affect (range *R* ^2^_adjusted_ = .01–.08). None of the main effects of the compassion measures nor their interaction terms with self‐blame and rumination were significant (all *p*_*adj*._ > .05). However, there were main effects of self‐blame on negative affect (models c and d), indicating a positive relationship of self‐blaming thoughts with internalizing symptoms, when accounting for compassion: (c) *ß* = .36, 95% CI [0.12, 0.60], *p*_adj._ = .014, and (d) *ß*_bootstrapped_ = .36, 95% CI [0.10, 0.61], *p*_adj._ = .008.

### 3.3 Research question 2: Quadratic associations

In a second step, we introduced quadratic terms for all empathy, compassion, and ToM measures to our regression models in interaction with negative thinking processes. Comparisons of model performance between linear and quadratic models yielded mixed results (see Supplementary Tables S5–S10).

Results showed that only the effect of (IRI perspective taking)^2^ on stress passed FDR correction, *ß* = .50, 95% CI [0.15, 0.86], *p*_adj._ = .030. Here, the quadratic fit performed better compared to the linear model (see Supplementary Table S7), indicating that lower and higher levels of self‐reported perspective taking were associated with higher stress levels. None of the other quadratic terms of empathy, compassion, or ToM explained stress or negative affect (all *p*_adj._ > .05).

For negative affect, neither the quadratic terms of empathy and ToM measures nor their interactions with negative thinking processes were significant (all *p*s > .05). The main effect of self‐blame on negative affect was still significant when including the EmpaToM valence contrast, its quadratic terms, and interactions with self‐blame and rumination, *ß* = .40, 95% CI [0.12, 0.69], *p*_adj._ = .032. Also, the effect of IRI personal distress on negative affect, *ß* = .49, 95% CI [0.26, 0.71], *p*_adj._ < .001, and stress, *ß* = .55, 95% CI [0.32, 0.78], *p*_adj._ < .001, remained stable when adding its quadratic term and interactions. Interestingly, when introducing the quadratic term of IRI perspective taking and its interactions with self‐blame to describe negative affect, the main effect of self‐blame (from step 1) was not significant. Still, there was a significant linear negative relationship between IRI perspective taking and negative affect, *ß*_bootstrapped_ = −.41, 95% CI [−0.60, −0.21], *p*_adj._ < .001, indicating an association of lower self‐reported perspective taking with more negative affect.

Because the main effects of models that include interaction terms are difficult to interpret and none of the interaction terms were significant, in a third step we removed the interactions, as suggested by Lorah (2020). This enabled us to explore whether the significant main effects of perspective taking and personal distress remained stable (see Supplementary Tables S11 and S12). However, the quadratic main effect of IRI perspective taking on stress showed not significant when removing the interaction terms from the model, *ß* = .29, 95% CI [−0.03, 0.62], *p*_adj._ = .174. We therefore refrain from any further interpretation. Yet, results yielded linear main effects of perspective taking and personal distress on internalizing symptoms.

### 3.4 Research question 3: Effects on the whole brain level

We conducted flexible factorial models to evaluate whether stress and negative affect interact with empathy‐ and ToM‐related brain activation (research question 3). None of the activation patterns for the interactions survived the correction threshold (FWE). Uncorrected statistical maps and activation peaks are displayed in the supplements (see Supplementary Table S2 and Figures S1–S5).

### 3.5 Research question 4: Effects of ROIs

#### 3.5.1 Linear regression models

To test whether negative thinking processes moderate the effects of empathy‐ and ToM‐related brain activity on internalizing symptoms, we extracted brain activation in a priori defined ROIs for the empathy contrast during the video epoch (emotional > neutral) as well as for the ToM contrast (ToM > nToM) during the video and the question epoch. The resulting beta estimates were used to describe stress and negative affect in interaction with negative thinking processes (see Table S4 in supplements for statistics).

##### Empathy‐related brain activation

**FIGURE 4:**
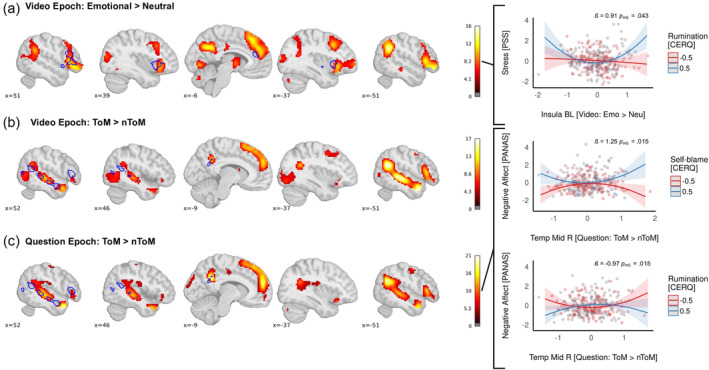
Brain activation derived from the (a) emotional > neutral contrast; (b) ToM > nToM contrast during the video epoch, and the (c) ToM > nToM contrast during the question epoch. Please note that the displayed contrasts were already reported, but only for a sample subset in Kanske et al. (2015) and two separate subsets of the sample in Tholen et al. (2020). Statistical maps are family wise error (FWE) corrected at *p* < .05 with a cluster‐extent threshold of *k* = 10 contiguous voxels. Blue circles indicate respective regions of interest. On the right side, interaction plots show quadratic relationships with respective brain activation and self‐blame versus rumination predicting stress and negative affect.

The one‐sample *t* test of the contrast emotional > neutral videos yielded a network of brain activations (see Table 3 and Figure 4) matching those previously observed in a subset of the described sample (Kanske et al., 2015).

**TABLE 3:** Activation peaks for empathy and ToM.

AAL label	H	Cluster ID	X	Y	Z	Peak stat	Cluster size (voxel)	Network
*Video epoch: emotional* > *neutral*								
Superior frontal gyrus, medial	L	4	−3	45	33	13.15	1621	DMN
Inferior frontal gyrus, pars orbitalis	L	2	−45	36	−6	13.88	1270	DMN
Inferior frontal gyrus, pars orbitalis	R	6	45	27	−3	12.32	966	DMN
Middle cingulate gyrus	L	10	0	−18	39	10.37	103	DMN
Thalamus^a^	R	11	6	0	0	8.63	337	‐
Middle temporal gyrus	L	13	−54	−30	−12	6.98	39	‐
Fusiform gyrus	R	15	27	−69	−9	5.73	51	VIS
Middle occipital gyrus	L	12	−39	−90	9	7.20	93	VIS
Supramarginal gyrus	L	1	−57	−51	33	16.31	650	DMN
Supramarginal gyrus	R	8	63	−48	27	11.21	690	DMN
Precuneus	L	5	−6	−60	33	13.06	673	DMN
Lingual gyrus	L	9	−6	−75	−3	10.39	274	VIS
Vermis 3		14	0	−48	−18	6.77	29	‐
Cerebellum (Crus I)	L	3	−18	−78	−33	13.21	219	‐
Cerebellum (Crus I)	R	7	21	−78	−33	11.88	170	‐
*Video epoch: ToM > nToM*								
Superior frontal gyrus, medial	L	4	−6	54	30	12.15	945	DMN
Inferior frontal gyrus, pars orbitalis	R	11	51	30	−9	6.42	69	DMN
Inferior frontal gyrus, pars triangularis	L	3	−51	24	6	12.28	320	DMN
Superior frontal gyrus	L	8	−42	6	51	8.69	167	FPN
Caudate nucleus	L	12	−12	6	12	5.31	15	‐
Temporal pole: middle temporal gyrus	R	6	54	12	−24	12.01	745	DMN
Precuneus	L	5	−6	−51	36	12.02	160	DMN
Angular gyrus	L	1	−54	−57	24	17.17	2023	DMN
Cerebellum (Crus I)	R	2	27	−78	−33	15.97	799	‐
Cerebellum (Crus II)	L	7	−24	−78	−36	11.91	105	‐
*Question epoch: ToM > nToM*								
Superior frontal gyrus, medial	L	6	−9	54	21	18.89	1587	‐
Inferior frontal gyrus, pars triangularis	L	7	−51	24	6	14.14	299	DMN
Inferior frontal gyrus, pars triangularis	R	9	54	30	0	7.74	85	FPN
Cerebral white matter^a^	R	17	21	36	0	5.39	12	‐
Insula	R	14	30	18	−18	5.71	10	DMN
Temporal pole: middle temporal gyrus	R	4	51	9	−33	19.77	1319	DMN
Posterior cingulate gyrus	L	2	−6	−51	33	20.20	387	DMN
Postcentral gyrus	L	10	−54	−6	48	7.72	57	SOM
Cerebral white matter^a^	R	12	18	−6	30	6.72	115	‐
Middle cingulate gyrus	L	8	0	−15	39	12.23	260	DMN
Precentral gyrus	R	13	36	−21	51	5.73	78	SOM
Precentral gyrus	R	15	42	−21	63	5.46	30	SOM
Paracentral lobule	L	16	−12	−30	54	5.42	11	‐
Postcentral gyrus	L	18	−18	−39	69	5.26	12	SOM
Angular gyrus	L	5	−51	−57	24	19.51	1496	DMN
Cuneus	L	11	−9	−93	30	7.70	218	VIS
Cerebellum (Crus I)	R	1	27	−78	−36	20.78	114	‐
Cerebellum (Crus II)	L	3	−27	−81	−36	20.01	93	‐

Abbreviations: AAL, automatic anatomic labeling; DMN, default mode network; FPN, frontoparietal network; H, hemisphere; SOM, somatomotor network; VIS, visual network.

^a^ Peak was outside the range of AAL Atlas; therefore, the labeling was based on the Desikan‐Killiany cortical atlas.

We conducted regression models to probe the association of stress and negative affect with brain activation of (a) the bilateral AI ROI and (b) the right IFG ROI. As an association between ACC activation and stress has not been consistently reported (Berretz et al., 2021), we calculated two separate models using the (c) right ACC ROI and (d) mPFC ROI to only describe negative affect, not stress. In general, IVs explained little variance of stress and negative affect (range *R* ^2^_adjusted_ = .02–.07).

Empathy‐related brain activation in these ROIs did not describe negative affect or stress directly nor in interaction with negative thinking processes. However, in all models that included the respective ROI activation as IVs, self‐reported self‐blaming thoughts described negative affect: (a) *ß* = .32, 95% CI [0.09, 0.56], *p*_adj._ = .029, (b) *ß* = .33, 95% CI [0.10, 0.57], *p*_adj._ = .023, (c) *ß* = .33, 95% CI [0.09, 0.56], *p*_adj._ = .027, (d) *ß* = .33, 95% CI [0.09, 0.56], *p*_adj._ = .026.

##### ToM‐related brain activation during video epoch

We conducted a one‐sample *t* test for the contrast ToM > nToM to derive brain activation during the video epoch (see Figure 4). Peak activations are displayed in Table 3 and are comparable to those detected by previous studies (Kanske et al., 2015).

We were interested in the association of stress (outcome 1) and negative affect (outcome 2) with the brain activation of (a) the right IFG ROI and (b) the middle temporal gyrus ROI based on the video epoch. As precuneus activation and negative affect have not been consistently linked (Lindquist et al., 2016), we described stress only, not negative affect, including brain activation of (c) the left precuneus ROI. Last, as the mPFC activity has been only associated with negative affect (Lindquist et al., 2016), we used the (d) mPFC ROI to only describe negative affect. The IVs generally explained little variance of stress and negative affect (range *R* ^2^_adjusted_ = .01–.07).

In these ROIs, ToM‐related brain activation based on the video epoch as a measure for more implicit ToM neither directly described stress or negative affect nor in interaction with negative thinking processes. However, self‐reported self‐blaming thoughts were significantly associated with negative affect in three models, including (a) IFG, *ß* = .32, 95% CI [0.09, 0.55] *p*_adj._ = .029, (b) temporal gyrus, *ß* = .33, 95% CI [0.09, 0.56], *p*_adj._ = .026, and (d) mPFC activation, *ß* = .33, 95% CI [0.10, 0.57], *p*_adj._ = .023.

##### ToM‐related brain activation during question epoch

For the question epoch, the one‐sample *t* test of the ToM > nToM contrast showed similar activation patterns compared to the video epoch. Peak activations can be found in Table 3.

None of the interactions of ToM‐related brain activation during the question epoch and negative thinking processes described stress or negative affect. The main effect of ToM‐related brain activity in the middle temporal gyrus was positively associated with stress, *ß* = .29, 95% CI [0.06, 0.51], *p*_adj._ = .047. Self‐reported self‐blaming thoughts were related to stress when including (c) temporal gyrus activation, *ß* = .31, 95% CI [0.07, 0.54], *p*_adj._ = .047, and negative affect when including (a) IFG, *ß* = .33, 95% CI [0.09, 0.57], *p*_adj._ = .025, (b) temporal gyrus activation, *ß* = .33, 95% CI [0.10, 0.56], *p*_adj._ = .023, and (d) mPFC activation, *ß* = .33, 95% CI [0.10, 0.56], *p*_adj._ = .022.

#### 3.5.2 Quadratic regression models

In a second step, we introduced quadratic terms of all ROI‐associated brain activation to the respective regression models in interaction with self‐blame and rumination. Overall, quadratic models yielded better performance when including empathy‐related ROIs compared to linear models (see Supplementary Tables S6 and S9). Regarding the quadratic models, including the ToM‐related ROIs, performance comparisons indicated only a better overall model fit in some cases compared to the linear models (see Supplementary Tables S7 and S10).

In the first step (without quadratic terms), the insula activation (derived from the emotional > neutral contrast) was neither associated with stress directly nor in interaction with self‐blame or rumination. However, adding the quadratic term of the insula activation yielded a better model fit compared to the linear model (see Supplementary Table S6): There was a significant main effect of (insula activation)^2^ on stress, *ß* = .43, 95% CI [0.11, 0.74], *p*_adj._ = .043, as well as a significant interaction effect of (insula activation)^2^ with rumination on stress, *ß* = .91, 95% CI [0.29, 1.53], *p*_adj._ = .043 (see Figure 4a).

For the ToM > nToM contrast derived from the video epoch, neither main nor interaction effects of quadratic terms of ROI brain activation with negative thinking processes were significantly associated with negative affect or stress.

However, for the ToM > nToM contrast during the question epoch, the quadratic regression models overall fitted better compared to the linear models (see Supplementary Table S10). Although temporal gyrus activation did not describe negative affect in the linear model, there were significant interaction effects when quadratic terms of the temporal gyrus ROI were introduced in the second step. The interactions of (middle temporal gyrus activation)^2^ × self‐blame, *ß*_bootstrapped_ = 1.25, 95% CI [0.40, 2.19], *p*_adj._ = .015, and (middle temporal gyrus activation)^2^ × rumination, *ß*_bootstrapped_ = −.97, 95% CI [−1.65, −0.33], *p*_adj._ = .015, were both significant with respect to negative affect in the same model (see Figure 4c). Other associations did not pass FDR correction.

## 4 DISCUSSION

Our primary research aim was to investigate how empathy, ToM, and negative thinking processes (specifically, rumination and self‐blame) relate to stress and negative affect. This research question was inspired by a theoretical framework (Tone & Tully, 2014), which proposes that negative thinking processes moderate the relationships of empathy and ToM with internalizing symptoms. To investigate this complex interplay of socio‐affective and ‐cognitive processes with internalizing symptoms, namely negative affect and stress, we used a multi‐method approach collecting self‐report, behavioral, and fMRI data. We tested our questions by reanalyzing the baseline data of a large‐scale, longitudinal mental training study, the ReSource project (Singer et al., 2016).

### 4.1 Research question 1: Linear associations

In contrast to our first assumption, we did not find that empathy, compassion, or ToM were associated with internalizing symptoms in interaction with negative thinking processes using linear regression analyses. Self‐reported personal (empathic) distress was positively associated with stress and negative affect, irrespective of self‐blame and rumination. This finding indicates that higher levels of self‐reported personal distress are generally associated with higher levels of internalizing symptoms and is in line with previous results focusing on depression in clinical and subclinical samples (Förster et al., 2022; Schreiter et al., 2013). Also, consistent with previous findings (Schreiter et al., 2013), there was no support for a negative association of compassion with internalizing symptoms and no interaction of compassion with self‐blame and rumination. As compassion is defined as a positive feeling for others (Singer & Klimecki, 2014), this finding is not surprising. It corresponds well with research on compassion trainings, showing strong associations with increased positive affect and activity in brain areas related to positive affect (Förster et al., 2022; Klimecki et al., 2014; Singer & Engert, 2019).

Interestingly, none of the behavioral measures significantly described stress and negative affect. This finding may be because the EmpaToM captures situational (state) empathy and ToM and thus may relate more strongly to situational negative affect and stress compared to prolonged states of stress and negative affect as measured with the PSS and PANAS. Also, previous literature found that empathy in daily life and its effects on well‐being vary across situational contexts (Depow et al., 2021). However, the association of situational empathy with prolonged internalizing symptoms and general mood states still has to be further explored.

### 4.2 Research question 2: Quadratic associations

In contrast to our expectations, we also found no significant quadratic effects of self‐reported or behavioral empathy, compassion, or ToM on internalizing symptoms that were moderated by negative thinking processes (and passed correction for multiple testing). However, there was a quadratic main effect of self‐reported ToM on stress in the regression model with—but not without—interaction terms. This result proved rather unstable and is reflected by inconsistencies in the research literature: While Tully et al. (2016) reported that lower and higher ToM was associated with depression, Powell (2018) found that lower and higher self‐reported ToM was associated with stress but not depression. The reason for the differences across these findings might be different sample characteristics (student sample vs. wider population), various conceptualizations of empathy or ToM (empathic concern and compassion vs. empathy vs. cognitive empathy), other uses of measures and outcome variables (e.g., depression vs. negative affect subscale) that assess different aspects of internalizing symptoms. Tone and Tully (2014) suggested that moderating influences of emotional dysregulation or negative thinking processes might vary across the lifespan. This idea could explain (in addition to differences in measurements) why we did not find moderating effects of negative thinking processes. Some of the negative thinking processes potentially moderate linear or nonlinear effects of self‐reported or behavioral empathy, compassion, and ToM on internalizing symptoms, but only at specific developmental stages.

In summary, we found no support for quadratic relationships between self‐reported or behaviorally assessed empathy, compassion, and ToM with internalizing symptoms. These results imply the need for replication studies as reports of a quadratic relationship are inconsistent and dependent on measures and samples. Yet, our exploratory analyses excluding the interaction terms showed linear relationships between self‐reported empathy and ToM with internalizing symptoms. Lower perspective taking and higher personal distress was associated with higher levels of stress and negative affect.

### 4.3 Research question 3: Effects on the whole brain level

We did not find support for different neural patterns for empathy and ToM dependent on individual profiles of perceived stress or negative affect at a whole brain level when using FWE correction. This finding could be due to the restricted variance of stress and negative affect in our sample. Future studies could investigate potential associations in samples with more variance and in clinical samples.

### 4.4 Research question 4: Effects of ROIs

We did not find linear interaction effects between empathy‐ and ToM‐related brain activity with negative thinking processes on internalizing symptoms. However, using nonlinear terms revealed three significant interactions that were robust enough to pass FDR correction.

We found that both low and high empathy‐ and compassion‐related insula activity, in interaction with rumination, was associated with more stress but not with more negative affect. Stress levels seemed average for those who reported less rumination, irrespective of empathy‐related insula activation. However, for people who reported more rumination, low and high empathy‐related insula activation was associated with higher perceived stress during the past weeks. The insula is not only related to interoceptive awareness but to general social–emotional functioning (Singer et al., 2009). As such, the insula's involvement spans a variety of emotions that arise particularly during social interactions, such as empathy and compassion (Singer et al., 2009) but also guilt, shame, and embarrassment (Bastin et al., 2016). Our results align with the expectation that “over‐empathizing” can, under specific circumstances, result in higher levels of empathic stress and, in prolonged or extreme forms, even in empathic distress fatigue (Klimecki & Singer, 2011). The CERQ rumination scale mainly captures rumination about one's own feelings. So, when participants who tend to recruit more empathy‐related insula function then ruminate about their own emotional state when sharing the affect of others, they might feel more stressed by the intensity of their own emotions. On the other hand, perceived stress was also high for people with high rumination levels who showed *lower* empathy‐related (compared to average) insula activation. Here, in people with lower empathy and a tendency to ruminate more, perceived stress could result from thinking about a discrepancy between one's own mental state (e.g., neutral) and the emotions of others (negative). This interpretation is in line with findings that hypoactivation in the insula has been associated with lower trait empathy and higher levels of alexithymia, a trait that is defined as having difficulties with the identification and description of feelings (Silani et al., 2008).

Concerning our results of quadratic associations of ToM‐related brain activation in the middle temporal gyrus, there are different aspects to discuss: First, we evaluate our results considering previous studies, and second, we try to explain the different patterns we observed for self‐blame and rumination.

Our results are broadly in line with the findings of Powell (2018), who reported that high and low ToM in people with high suppression tendencies were associated with anxiety. Even though we did not find quadratic associations using self‐report data, we found a quadratic association of ToM‐related brain activation on the PANAS negative affect subscale that was self‐blame dependent. The negative affect subscale particularly captures anxiety‐related emotions and thus corresponds to Powell's (2018) findings. Here, *low* and *high* ToM‐related brain activation in the middle temporal gyrus was associated with more negative affect, but only in those with *higher* levels of self‐blame. Lower versus higher brain activation in the middle temporal gyrus during the question epoch might be cautiously interpreted as "under‐" versus "over‐mentalizing". People who tend to over‐mentalize do not necessarily infer other people's mental states correctly (Sharp et al., 2011). Combined with more self‐blame behavior, this could result in negative affect, such as guilt, shame, or anger (as captured by the PANAS). Also, tendencies to under‐mentalize could cause a variety of adverse effects, particularly high self‐blame behavior, as these individuals might also make prediction errors regarding how other people will behave (Montag et al., 2011).

Second, within the same regression model, *low* and *high* ToM‐related activity in the middle temporal gyrus was associated with more negative affect, but only in those with *lower* rumination. At first glance, this effect seems contradictory to the first effect, as we would have expected this pattern in people with *high* rumination. However, it corroborates the importance of differentiating between specific negative thinking processes, as they appear to have differential effects on the association between ToM and internalizing symptoms. Looking closer, one might cautiously speculate that when people who tend to ruminate less than average are put in a situation where they need to explicitly use ToM abilities (as within the EmpaToM question epoch), they are forced to think about other people's negative emotional states. As they might not be used to this, this could result in more negative affect. In contrast, people who ruminate more may already be used to “mentalizing” about others and their own emotional states, which does not result in relatively more negative affect when they are asked to use their mentalizing or ToM skills.

Of course, these interpretations must be treated with great caution. Another explanation for this unexpected result could be that the effect is a false positive finding. When looking at bivariate correlations (see correlation matrix in Figure 3), there was no significant correlation of rumination with negative affect or stress, nor with any ROI activation. In contrast, self‐blame was positively associated with both stress and negative affect. Moreover, the difference between people with low and high middle temporal gyrus activity in people with low versus high self‐blame seems to be larger compared to those with low versus high rumination. As the third interaction plot displays, confidence bands overlap more for low versus high rumination, indicating a higher uncertainty for this contrast (see Figure 4c).

Finally, this uncertainty and the results could be driven by the nature of the ROI itself. To minimize the size of the ROIs, we carefully restricted the regions by using conjunctions of areas that are associated with empathy, ToM, and internalizing symptoms. Nevertheless, the conjunction of the ToM‐related functional mask (Bzdok et al., 2012) with the structural mask of the middle temporal gyrus still resulted in a relatively large area spanning from the temporal pole toward the temporo‐parietal junction. Therefore, future studies could investigate differential effects of specific temporal regions on internalizing symptoms.

### 4.5 Limitations

Despite a large sample and a validated task, several limitations must be noted. First, our sample is relatively homogeneous, with rather high education status, a general interest in mindfulness, and depression scores below a clinical cut‐off. The sample characteristics restrict generalization to other samples. On the other hand, it covers a broader age range than previous studies in the field. Also, there are some caveats to the approach of examining stress and negative affect as subclinical internalizing symptoms. Because the depression sum score had limited variance in our sample, we decided not to use it as an outcome. Instead, we used stress and negative affect as subclinical proxies for internalizing psychopathology, which was supported by significant positive correlations with the BDI II (Beck et al., 1996). In line with this, previous research linked elevated perceived stress and subthreshold depression to an increased risk of depression onset (Cristóbal‐Narváez et al., 2022; Cuijpers, 2004). Moreover, depression sum scores in particular aggregate highly heterogeneous symptoms, thereby masking interindividual variance (Fried et al., 2014). Focusing on isolated symptoms and using a dimensional approach allows for the exploration of more detailed risk combinations in non‐clinical samples. However, we recognize that the line between elevated stress and negative affect, which are warning signs of internalizing disorders, and common short‐term responses to daily stressors is not always clear (Cooper, 2013).

Moreover, the rumination and self‐blame subscales of the CERQ had relatively low internal consistencies. However, the CERQ is generally a well‐validated, commonly used measure (Betegón et al., 2022; Garnefski & Kraaij, 2007; Loch et al., 2011). In addition, it should be noted that the quadratic fit of the models did not perform better for all models, indicating that, depending on the measure, a linear fit provided a more appropriate description of the data (see Supplementary Tables S5–S10). Also, the reported main effects of the regression models that included interaction terms should not be interpreted in isolation. This is why we further explored significant quadratic main effects of empathy and ToM without the interaction terms, which yielded null results.

Due to our cross‐sectional analysis, we cannot draw any conclusions about causal associations between empathy, ToM, and internalizing symptoms. Even though we considered interactions, we still looked at these processes in isolation, so associations between brain areas or interactions of empathy and ToM could also be altered and contribute to the complex interplay of processes we investigated here.

## 5 CONCLUSION

This study investigated the interplay of empathy, compassion, ToM, and negative thinking processes (self‐blame and rumination) with internalizing symptoms (stress and negative affect). To disentangle these processes, we employed a multi‐method approach including neural, self‐report, and behavioral data in the context of a healthy baseline sample of a large‐scale mental training study, the ReSource project.

Failing to support our expectations, negative thinking processes moderated neither the association of self‐reported nor behaviorally assessed empathy, compassion, and ToM with internalizing symptoms. Also, we did not find quadratic associations of the behaviorally assessed socio‐affective and cognitive processes with internalizing symptoms. However, both lower self‐reported ToM and higher empathic distress were associated with higher levels of internalizing symptoms. Additionally, by incorporating fMRI data, we found that both lower and higher, compared to average, empathy‐related levels of brain activation in the AI was associated with stress but only for people high in rumination. Also, the association between activation in the middle temporal gyrus during explicit ToM performance and negative affect was quadratic and a function of specific levels of rumination and self‐blame.

Our findings advance current research by helping to identify which complex risk combinations of socio‐affective and ‐cognitive functioning are relevant to internalizing symptoms in a wider sample. Moreover, our results on quadratic relationships highlight the importance of a nuanced exploration of individual differences and interactions when examining risk combinations. Future interventions could target these risk constellations to strengthen mental health.

## AUTHOR CONTRIBUTIONS

Tania Singer initiated and developed the ReSource Project, model, and training protocol and secured all funding. Anne Böckler‐Raettig, Fynn‐Mathis Trautwein, and Philipp Kanske were involved in the data assessment. Annika C. Konrad developed the research questions and the statistical analysis plan under the supervision of Jason Stretton, Katharina Förster, Philipp Kanske, and Tim Dalgleish; Fynn‐Mathis Trautwein preprocessed the fMRI data. Annika C. Konrad analyzed the data. Annika C. Konrad drafted the article, and all other co‐authors contributed to writing the manuscript and approved its final version for submission.

## FUNDING INFORMATION

This study forms part of the ReSource Project (headed by T.S.). Data for this project was collected between 2013 and 2016 at the former Department of Social Neuroscience at the MPI CBS Leipzig. T.S. (Principal Investigator) received funding for the ReSource Project from the European Research Council (ERC) under the European Community's Seventh Framework Program (FP7/2007–2013), ERC grant agreement number 205557. Philipp Kanske was supported by grants from the German Research Foundation KA4412/2‐1, KA4412/4‐1, KA4412/5‐1, KA4412/9‐1, CRC940/CO7, IRTG2773/P4. Annika Konrad was supported by the Saxon Scholarship Program and the German Research Foundation KA4412/9‐1. The funding organization had no role in the design and conduct of the study, in the collection, management, analysis, and interpretation of the data, in the preparation of the manuscript, or in the decision to submit the manuscript for publication.

## CONFLICT OF INTEREST STATEMENT

The authors declare no competing interests.

## Supporting information


**DATA S1:** Supporting Information.

## Data Availability

In line with new data regulations (General Data Protection Regulation, GDPR), we regret that our data cannot be shared because we did not obtain explicit participant agreement for data sharing with parties outside the Max Planck Institute Leipzig. The code supporting this study's findings is available from the corresponding author upon request.
