# Supplementary material for: Risk factors for internalizing symptoms: The influence of empathy, theory of mind, and negative thinking processes
Source: Hum Brain Mapp. 2024 Feb 24;45(3):e26576. doi: 10.1002/hbm.26576 (PMC10893974; doi:10.1002/hbm.26576)
Supplement: Supplementary file 1 — DATA S1: Supporting Information. [file HBM-45-e26576-s001.docx]

Supplementary materials

# Sample differences with other ReSource studies

Three studies used behavioral or neural data of the task measuring empathy and ToM (EmpaToM task; Kanske et al., 2015; Tholen et al., 2020; Trautwein et al., 2020): Kanske et al. (2015) validated the EmpaToM by using behavioral and neural EmpaToM data from *N* = 178 ReSource participants (sample 1). Tholen et al. (2020) applied an fMRI item analysis using neural and behavioral EmpaToM data in two separate ReSource samples (sample 1: *N* = 178, sample 2: *N* = 130). Trautwein et al. (2020) used only the behavioral EmpaToM data of samples 1 and 2 combined (*N* = 308). We also combined both samples, but we excluded an additional *n* = 6 participants compared to these three studies. As we used an updated version of the software (SPM12, not SPM8), the results of the first level analysis slightly differed. Compared to Tholen et al. (2020), we had problems estimating degrees of freedom at the first level in *n* = 6 cases and excluded those cases from further analysis. Thus, the results of the baseline EmpaToM data differ slightly from previous studies and rely on 302 participants. Neural EmpaToM results vary from Kanske et al. (2015) and Tholen et al. (2020), as we combined samples 1 and 2 and further excluded incomplete datasets of relevant self-report covariates (negative affect and stress) in the whole brain analyses. For all analyses, including the Perceived Stress Scale (Cohen et al., 1983), *n* = 6 incomplete cases were excluded; for all analyses, including the Positive and Negative Affect Scale (Krohne et al., 1996), there were *n* = 5 incomplete cases.

| Table S1  Comparing EmpaToM datasets across different ReSource project studies | | |
| --- | --- | --- |
| **Paper** | ***N* (Baseline Data T0)** | **Aim** |
| Kanske et al., 2015 | - Sample 1: *N* = 178 | Validation EmpaToM task using behavioral and neural EmpaToM data |
| Tholen et al., 2020 | Separate analysis^a,b^:   - Sample 1: *N* = 178 - Sample 2: *N* = 130 | Investigation of fMRI item analyses of the neural EmpaToM data |
| Trautwein et al., 2020 | - Sample 1 and Sample 2 combined: *N* = 308^a^ | Investigation of differential effects of mental trainings by using behavioral measures of EmpaToM and change scores |
| Konrad et al., 2023 | - Sample 1 and sample 2 combined: *N* = 302 | Investigation of the interplay between behavioral and neural EmpaToM data with negative thinking processes and internalizing symptoms |
| *Note.* ^a^Compared to Tholen et al. (2020) and Trautwein et al. (2020), we excluded *n* = 6 additional participants. Also, incomplete cases for questionnaire data yielded different sample sizes in the respective whole brain and regression analyses. ^b^Compared to Tholen et al. (2020), we combined samples 1 and 2 in our analysis. | | |

# Sensitivity analysis

To determine the minimum detectable effect based on our sample and models, we used the G*Power sensitivity analysis tool for linear multiple regression (fixed model) with α = 0.05 (two-tailed), β = 0.2 (assumed power = 0.8), given the sample size N = 296 and seven predictors for the linear models:

Y = b_0_ + b_1_ CV_1_ + b_2_ CV_2_ + b_3_ MV_1_ + b_4_ MV_2_ + b_5_ IV + b_6_ IV x MV_1_ + b_7_ IV x MV_2_ + ε

Please note that IV stands for independent variable, which resembles the respective ToM or empathy-related measure (e.g., self-reported personal distress), MV for moderating variable (MV_1_ = self-blame, MV_2_ = rumination), and CV for control variable (CV_1_ = age, CV_2_ = gender). The sensitivity analyses yielded a critical *t* = 1.97 (df = 288) and a minimum detectable effect size of *f^2^* = 0.027 (*d* = 0.32), suggesting that our sample size was large enough to detect small effects.

For the quadratic fit, we added the quadratic term of the respective independent variable (empathy or ToM measure) and two additional interaction terms (… + b_8_ IV_2_ + b_9_ IV^2^ x MV_1_ + b_10_ IV^2^ x MV_2_): With ten predictors, this resulted in a critical *t* = 1.97 (df = 285) and a minimum detectable effect size of *f^2^* = 0.027 (*d* = 0.32).

# Results from whole brain analysis

| Table S2  Activation Peaks of Flexible Factorial Models | | | | | | | | |
| --- | --- | --- | --- | --- | --- | --- | --- | --- |
| **AAL Label** | **H** | **Cluster ID** | **X** | **Y** | **Z** | **Peak Stat** | **Cluster Size (voxel)** | **Network** |
| **Question epoch: nToM x Stress > ToM x Stress** | | | | | | | | |
| Cerebral white matter^a^ | R | 1 | 21 | -27 | 30 | 3.98 | 14 | - |
| **Question epoch: ToM x Stress > nToM x Stress** | | | | | | | | |
| Superior frontal gyrus | R | 4 | 18 | 54 | 36 | 4.14 | 38 | DMN |
| Inferior frontal gyrus, pars triangularis | R | 5 | 57 | 27 | 24 | 3.79 | 42 | FPN |
| Inferior frontal gyrus, pars opercularis | L | 9 | -45 | 18 | 33 | 3.45 | 13 | FPN |
| Middle frontal gyrus | R | 2 | 30 | 21 | 60 | 4.26 | 44 | FPN |
| Caudate nucleus | R | 7 | 18 | 15 | 6 | 3.55 | 11 | - |
| Middle temporal gyrus | L | 8 | -51 | -42 | 6 | 3.48 | 11 | DMN |
| Middle temporal gyrus | R | 3 | 60 | -54 | 3 | 4.20 | 25 | DAN |
| Precuneus | R | 6 | 6 | -54 | 33 | 3.61 | 13 | DMN |
| Angular gyrus | R | 1 | 48 | -63 | 36 | 4.40 | 85 | DMN |
| **Question epoch: nToM x Negative Affect > ToM x Negative Affect** | | | | | | | | |
| Cerebral white matter^a^ | R | 3 | 18 | -24 | 33 | 3.84 | 24 | - |
| Precuneus | R | 1 | 18 | -51 | 21 | 4.23 | 30 | - |
| Middle occipital gyrus | L | 2 | -39 | -81 | 27 | 4.21 | 19 | DAN |
| **Video epoch: Neutral x Stress > Emotional x Stress** | | | | | | | | |
| Postcentral gyrus | R | 1 | 54 | -6 | 36 | 3.51 | 40 | SOM |
| **Video epoch: Neutral x Negative Affect > Emotional x Negative Affect** | | | | | | | | |
| Superior frontal gyrus | L | 2 | -21 | 54 | 0 | 3.63 | 15 | FPN |
| Middle frontal gyrus | R | 1 | 33 | 48 | 12 | 3.89 | 90 | FPN |
| Postcentral gyrus | R | 3 | 60 | -18 | 33 | 3.35 | 10 | DAN |
| *Note.* ^a^Peak was outside the range of the automatic anatomic labeling (AAL) Atlas; therefore, the labeling was based on the Desikan-Killiany cortical atlas. Abbreviations: H = Hemisphere; SOM = Somatomotor Network; DAN = Dorsal Attention Network; DMN = Default Mode Network; FPN = Frontoparietal Network. | | | | | | | | |

##

| 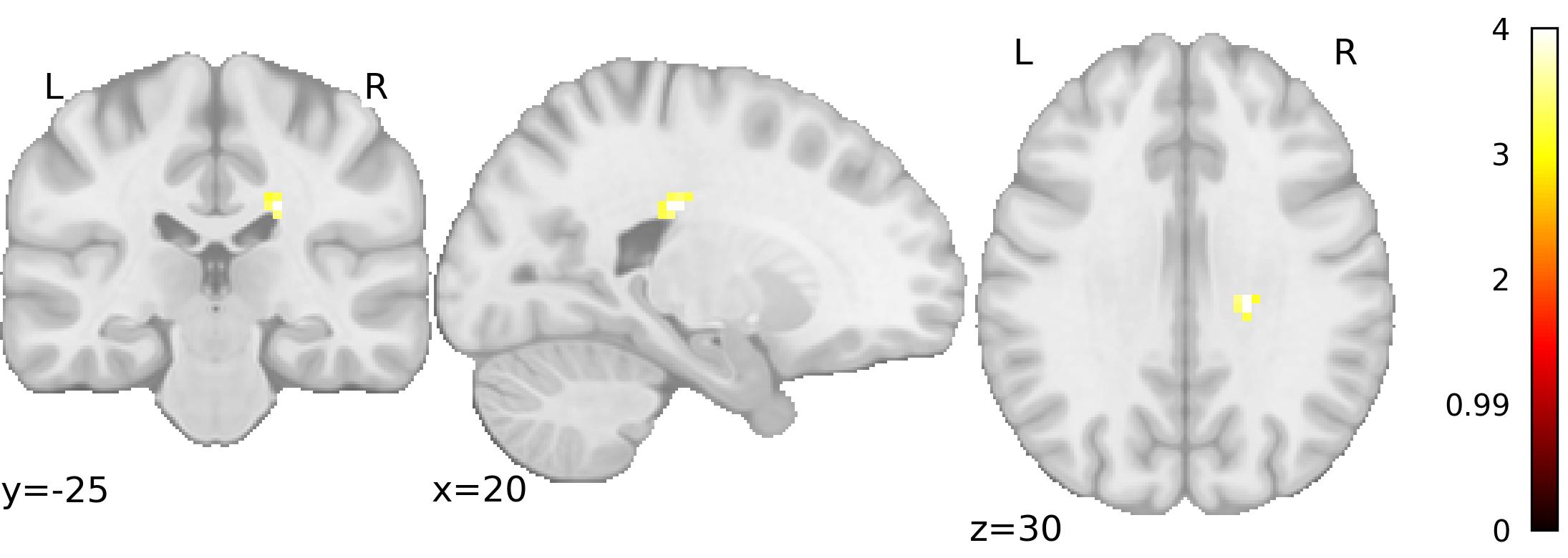 |
| --- |
| Figure S1. Brain activation for the contrast nToM x stress > ToM x stress during question epoch with a cluster threshold of k = 10 voxels |

| 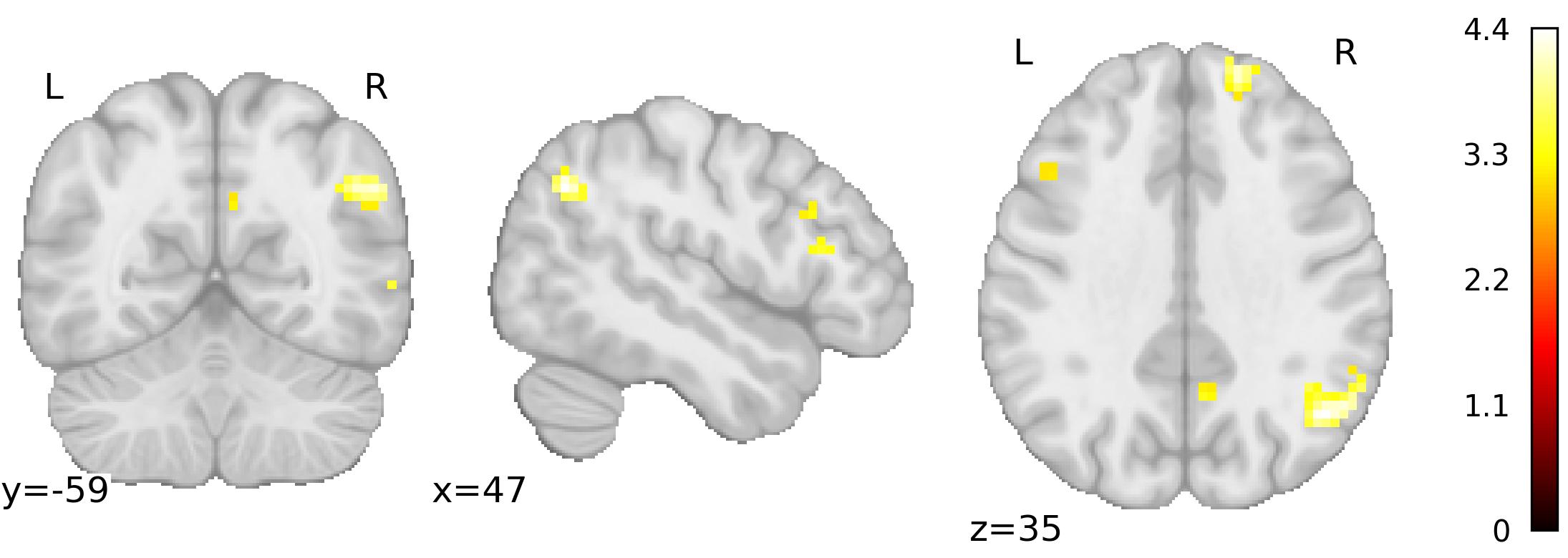 |
| --- |
| Figure S2. Brain activation for the contrast ToM x stress > nToM x stress during question epoch with a cluster threshold of k = 10 voxels |

| 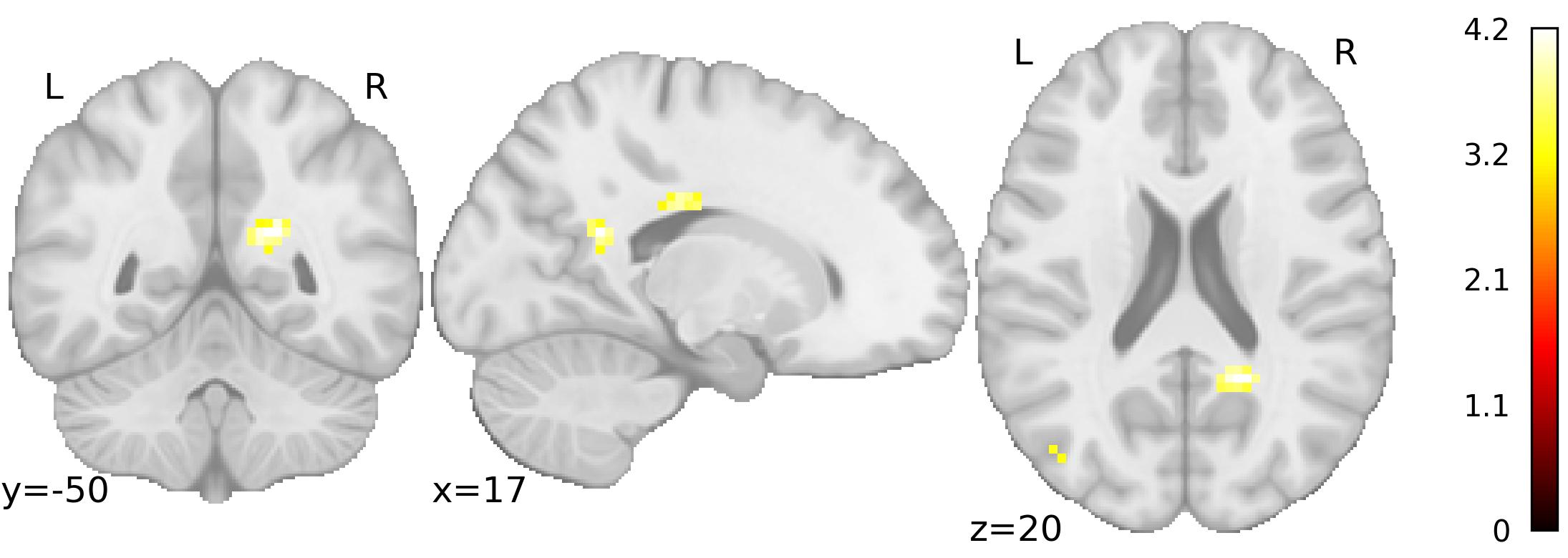 |
| --- |
| Figure S3. Brain activation for the contrast nToM x negative affect > ToM x negative affect during question epoch with a cluster threshold of k = 10 voxels |

| 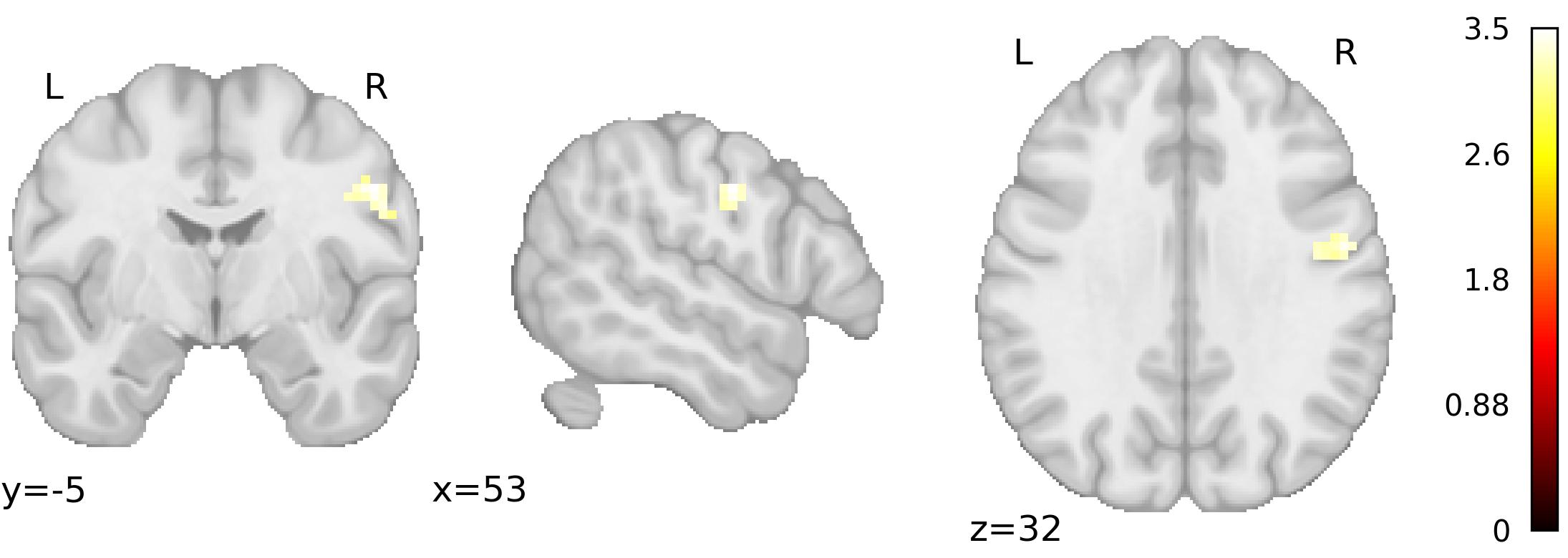 |
| --- |
| Figure S4. Brain activation for the contrast neutral x stress > emotional x stress during video epoch with a cluster threshold of k = 10 voxels |

| 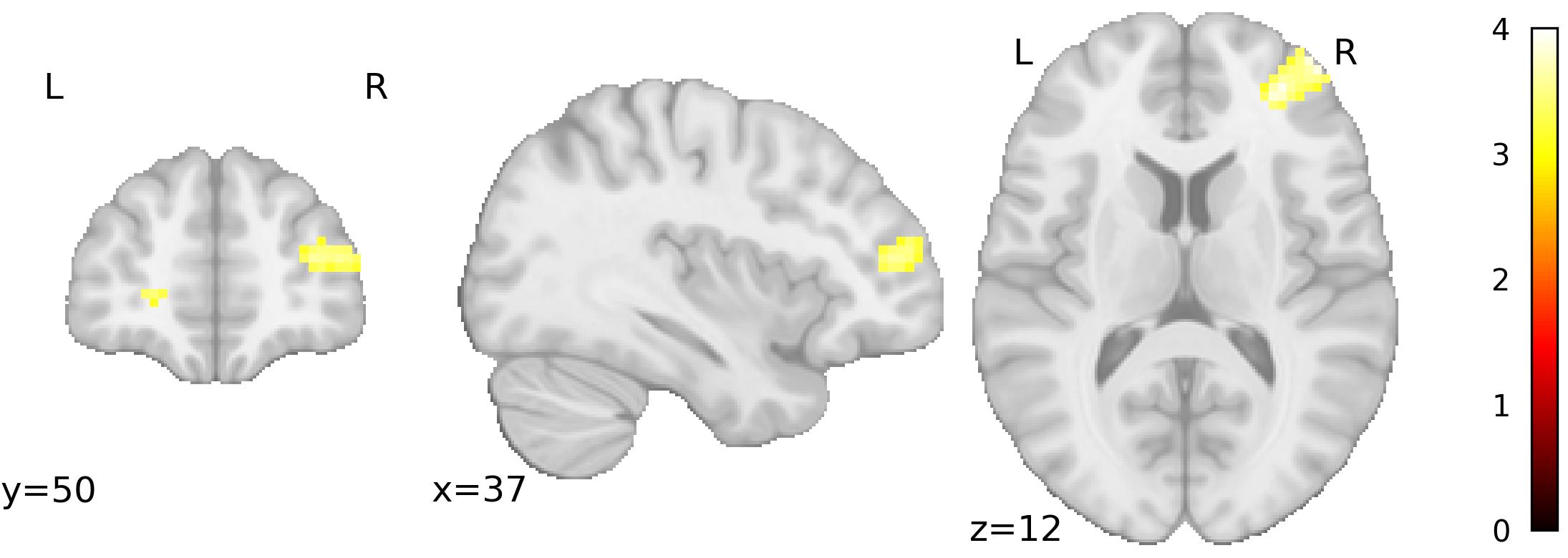 |
| --- |
| Figure S5. Brain activation for the contrast neutral x negative affect > emotional x negative affect during video epoch with a cluster threshold of k = 10 voxels |

# Results from regions of interest analysis

| Table S3  A priori defined regions of interest | | | | |
| --- | --- | --- | --- | --- |
| **Region** | **AAL Label** | **MNI [x y z]** (Bzdok et al., 2012) | **Associated with** | |
|  |  |  | **Negative affect**  (Lindquist et al., 2016) | **Stress**  (Berretz et al., 2021) |
| **Empathy-related regions of interest** | | | | |
| Amygdala | Amygdala_R | [22 -2 -16] | x | x |
| Anterior insula | Insula_R | [50 12 -8] | x | x |
|  | Insula_L | [-30 20 4] | x | x |
| Inferior frontal gyrus (pars triangularis) | Frontal_Inf_Tri_R | [50 30 4] | x | x |
| Anterior cingulate cortex | Cingulum_Ant_L | [-2 28 20] | x | - |
| Medial prefrontal cortex | Frontal_Sup_Medial_L | [-8 54 34] | x | - |
| **ToM-related regions of interest** | | | | |
| Inferior frontal gyrus  (pars triangularis) | Frontal_Inf_Tri_R | [54 28 6] | x | x |
| Right Middle temporal gyrus | Temporal_Mid_R | [52 -18 -12] | x | x |
| Precuneus | Precuneus_L | [2 -56 30] | - | x |
| Medial prefrontal cortex | Frontal_Sup_Medial_L | [-8 56 30] | x | - |
| *Note.* Please note that the associations of regions of interest with negative affect include regions that are valence general (associated with both negative and positive affect). | | | | |

| Table S4  Statistics table for regions of interest per contrast | | | | | |
| --- | --- | --- | --- | --- | --- |
| **Region** | **H** | **Contrast Value** | ***T* statistic** | ***p*** | ***p_corr._*** |
| **Video epoch: emotional > neutral** | | | | | |
| Amygdala | R | 0.00 | 0.31 | 0.378 | 0.907 |
| Insulae | BL | 0.07 | 4.35 | < .001 | < .001 |
| Inferior Frontal Gyrus  (pars triangularis) | R | 0.18 | 9.92 | < .001 | < .001 |
| Anterior cingulate cortex | L | 0.07 | 3.03 | .001 | .007 |
| Medial prefrontal cortex | L | 0.19 | 8.77 | < .001 | < .001 |
| **Video epoch: ToM > nToM** | | | | | |
| Inferior frontal gyrus  (pars triangularis) | R | 0.10 | 4.72 | < .001 | < .001 |
| Middle temporal gyrus | R | 0.08 | 7.52 | < .001 | < .001 |
| Precuneus | L | 0.07 | 5.06 | < .001 | < .001 |
| Medial prefrontal cortex | L | 0.26 | 9.56 | < .001 | < .001 |
| **Question epoch: ToM > nToM** | | | | | |
| Inferior frontal gyrus  (pars triangularis) | R | 0.14 | 5.22 | < .001 | < .001 |
| Middle temporal gyrus | R | 0.09 | 5.41 | < .001 | < .001 |
| Precuneus | L | 0.30 | 14.17 | < .001 | < .001 |
| Medial prefrontal cortex | L | 0.74 | 16.99 | < .001 | < .001 |
| *Note.* Abbreviations: H = Hemisphere; corr. = Bonferroni corrected | | | | | |

# Results from model fit comparison

| Table S5  Stress models with Compassion measures as independent variables | | | | | | | | | |
| --- | --- | --- | --- | --- | --- | --- | --- | --- | --- |
| Model Fit |  | **R^2^** | **R^2^ adj.** | **RMSE** | **Sigma** | **AIC^a^** | **AICc^a^** | **BIC^a^** | **Performance Score** |
| IRI Empathic Concern | | | | | | | | | |
| Quadratic |  | 0.05 | 0.02 | 0.96 | 0.97 | 0.54 | 0.48 | 0.00 | 0.71 |
| Linear |  | 0.03 | 0.01 | 0.97 | 0.98 | 0.46 | 0.52 | 1.00 | 0.29 |
| EmpaToM Compassion Rating | | | | | | | | | |
| Quadratic |  | 0.06 | 0.03 | 0.97 | 0.99 | 0.63 | 0.57 | 0.01 | 0.86 |
| Linear |  | 0.04 | 0.01 | 0.98 | 0.99 | 0.37 | 0.43 | 0.99 | 0.14 |
| *Notes.* All models include interaction terms of the independent variable with self-blame and rumination respectively; green indicates better performance of the quadratic versus linear model fit; abbreviations: AIC = Akaike's Information Criterion; AICc: Second-order (or small sample) Akaike's Information Criterion with a correction for small sample sizes; BIC = Bayesian Information Criterion; RMSE = Root Mean Squared Error; Sigma = Residual Standard Deviation; ^a^ weights. | | | | | | | | | |

| Table S6  Stress models with Empathy measures as independent variables | | | | | | | | | |
| --- | --- | --- | --- | --- | --- | --- | --- | --- | --- |
| Model Fit |  | **R^2^** | **R^2^ adj.** | **RMSE** | **Sigma** | **AIC^a^** | **AICc^a^** | **BIC^a^** | **Performance Score** |
| IRI Personal Distress | | | | | | | | | |
| Linear |  | 0.11 | 0.09 | 0.93 | 0.94 | 0.92 | 0.94 | 1.00 | 0.71 |
| Quadratic |  | 0.11 | 0.08 | 0.92 | 0.94 | 0.08 | 0.06 | 0.00 | 0.29 |
| EmpaToM Valence Rating | | | | | | | | | |
| Linear |  | 0.07 | 0.04 | 0.95 | 0.96 | 0.95 | 0.96 | 1.00 | 0.71 |
| Quadratic |  | 0.07 | 0.04 | 0.95 | 0.97 | 0.05 | 0.04 | 0.00 | 0.29 |
| Video: Emotional > Neutral Insula | | | | | | | | | |
| Quadratic |  | 0.09 | 0.06 | 0.94 | 0.96 | 0.97 | 0.96 | 0.12 | 0.86 |
| Linear |  | 0.05 | 0.02 | 0.96 | 0.97 | 0.03 | 0.04 | 0.88 | 0.14 |
| Video: Emotional > Neutral IFG | | | | | | | | | |
| Quadratic |  | 0.07 | 0.04 | 0.95 | 0.96 | 0.72 | 0.67 | 0.01 | 0.86 |
| Linear |  | 0.04 | 0.02 | 0.96 | 0.97 | 0.28 | 0.33 | 0.99 | 0.14 |
| *Notes.* All models include interaction terms of the independent variable with self-blame and rumination respectively; green indicates better performance of the quadratic versus linear model fit; orange indicates better performance of the linear versus quadratic model fit; abbreviations: AIC = Akaike's Information Criterion; AICc: Second-order (or small sample) Akaike's Information Criterion with a correction for small sample sizes; BIC = Bayesian Information Criterion; RMSE = Root Mean Squared Error; Sigma = Residual Standard Deviation; ^a^ weights. | | | | | | | | | |

| Table S7  Stress Models with ToM Measures as Independent Variables | | | | | | | | | |
| --- | --- | --- | --- | --- | --- | --- | --- | --- | --- |
| Model Fit |  | **R^2^** | **R^2^ adj.** | **RMSE** | **Sigma** | **AIC^a^** | **AICc^a^** | **BIC^a^** | **Performance Score** |
| IRI Perspective Taking | | | | | | | | | |
| Quadratic |  | 0.11 | 0.08 | 0.93 | 0.94 | 0.87 | 0.84 | 0.03 | 0.86 |
| Linear |  | 0.08 | 0.06 | 0.94 | 0.96 | 0.13 | 0.16 | 0.97 | 0.14 |
| EmpaToM ToM Performance | | | | | | | | | |
| Linear |  | 0.04 | 0.02 | 0.96 | 0.98 | 1.00 | 1.00 | 1.00 | 0.86 |
| Quadratic |  | 0.05 | 0.01 | 0.97 | 0.99 | 0.00 | 0.00 | 0.00 | 0.14 |
| Video: ToM > nToM IFG | | | | | | | | | |
| Linear |  | 0.03 | 0.01 | 0.97 | 0.98 | 0.83 | 0.86 | 1.00 | 0.71 |
| Quadratic |  | 0.04 | 0.01 | 0.96 | 0.98 | 0.17 | 0.14 | 0.00 | 0.29 |
| Question: ToM > nToM IFG | | | | | | | | | |
| Quadratic |  | 0.06 | 0.02 | 0.95 | 0.97 | 0.28 | 0.23 | 0.00 | 0.57 |
| Linear |  | 0.04 | 0.02 | 0.96 | 0.97 | 0.72 | 0.77 | 1.00 | 0.43 |
| Video: ToM > nToM Precuneus | | | | | | | | | |
| Linear |  | 0.04 | 0.01 | 0.98 | 0.99 | 0.86 | 0.88 | 1.00 | 0.71 |
| Quadratic |  | 0.05 | 0.01 | 0.97 | 0.99 | 0.14 | 0.12 | 0.00 | 0.29 |
| Question: ToM > nToM Precuneus | | | | | | | | | |
| Linear |  | 0.04 | 0.02 | 0.98 | 0.99 | 0.87 | 0.90 | 1.00 | 0.71 |
| Quadratic |  | 0.05 | 0.01 | 0.97 | 0.99 | 0.13 | 0.10 | 0.00 | 0.29 |
| Video: ToM > nToM Temporal Gyrus | | | | | | | | | |
| Quadratic |  | 0.05 | 0.02 | 0.96 | 0.98 | 0.37 | 0.31 | 0.00 | 0.57 |
| Linear |  | 0.03 | 0.01 | 0.97 | 0.98 | 0.63 | 0.69 | 1.00 | 0.43 |
| Question: ToM > nToM Temporal Gyrus | | | | | | | | | |
| Linear |  | 0.06 | 0.03 | 0.95 | 0.97 | 0.81 | 0.84 | 1.00 | 0.71 |
| Quadratic |  | 0.07 | 0.03 | 0.95 | 0.97 | 0.19 | 0.16 | 0.00 | 0.29 |
| *Notes.* All models include interaction terms of the independent variable with self-blame and rumination respectively; green indicates better performance of the quadratic versus linear model fit; orange indicates better performance of the linear versus quadratic model fit; abbreviations: AIC = Akaike's Information Criterion; AICc: Second-order (or small sample) Akaike's Information Criterion with a correction for small sample sizes; BIC = Bayesian Information Criterion; RMSE = Root Mean Squared Error; Sigma = Residual Standard Deviation; IFG = Inferior Frontal Gyrus; ^a^ weights. | | | | | | | | | |

| Table S8  Negative Affect Models with Compassion Measures as Independent Variables | | | | | | | | | |
| --- | --- | --- | --- | --- | --- | --- | --- | --- | --- |
| Model Fit |  | **R^2^** | **R^2^ adj.** | **RMSE** | **Sigma** | **AIC^a^** | **AICc^a^** | **BIC^a^** | **Performance Score** |
| IRI Empathic Concern | | | | | | | | | |
| Quadratic |  | 0.10 | 0.07 | 0.94 | 0.96 | 0.24 | 0.20 | 0.00 | 0.57 |
| Linear |  | 0.09 | 0.06 | 0.94 | 0.96 | 0.76 | 0.80 | 1.00 | 0.43 |
| EmpaToM Compassion Rating | | | | | | | | | |
| Quadratic |  | 0.13 | 0.10 | 0.92 | 0.94 | 0.83 | 0.79 | 0.02 | 0.86 |
| Linear |  | 0.10 | 0.08 | 0.94 | 0.95 | 0.17 | 0.21 | 0.98 | 0.14 |
| *Notes* All models include interaction terms of the independent variable with self-blame and rumination respectively; green indicates better performance of the quadratic versus linear model fit; abbreviations: AIC = Akaike's Information Criterion; AICc: Second-order (or small sample) Akaike's Information Criterion with a correction for small sample sizes; BIC = Bayesian Information Criterion; RMSE = Root Mean Squared Error; Sigma = Residual Standard Deviation; ^a^ weights. | | | | | | | | | |

| Table S9  Negative Affect Models with Empathy Measures as Independent Variables | | | | | | | | | |
| --- | --- | --- | --- | --- | --- | --- | --- | --- | --- |
| Model Fit |  | **R^2^** | **R^2^ adj.** | **RMSE** | **Sigma** | **AIC^a^** | **AICc^a^** | **BIC^a^** | **Performance Score** |
| IRI Personal Distress | | | | | | | | | |
| Quadratic |  | 0.16 | 0.13 | 0.91 | 0.92 | 0.21 | 0.17 | 0.00 | 0.57 |
| Linear |  | 0.15 | 0.13 | 0.91 | 0.93 | 0.79 | 0.83 | 1.00 | 0.43 |
| EmpaToM Valence Rating | | | | | | | | | |
| Linear |  | 0.09 | 0.06 | 0.94 | 0.96 | 0.84 | 0.87 | 1.00 | 0.71 |
| Quadratic |  | 0.09 | 0.06 | 0.94 | 0.96 | 0.16 | 0.13 | 0.00 | 0.29 |
| Video: Emotional > Neutral Insula | | | | | | | | | |
| Quadratic |  | 0.13 | 0.10 | 0.92 | 0.94 | 0.97 | 0.96 | 0.12 | 0.86 |
| Linear |  | 0.09 | 0.06 | 0.94 | 0.96 | 0.03 | 0.04 | 0.88 | 0.14 |
| Video: Emotional > Neutral IFG | | | | | | | | | |
| Quadratic |  | 0.10 | 0.07 | 0.94 | 0.95 | 0.39 | 0.33 | 0.00 | 0.57 |
| Linear |  | 0.09 | 0.06 | 0.94 | 0.96 | 0.61 | 0.67 | 1.00 | 0.43 |
| Video: Emotional > Neutral ACC | | | | | | | | | |
| Quadratic |  | 0.11 | 0.08 | 0.93 | 0.95 | 0.63 | 0.57 | 0.01 | 0.86 |
| Linear |  | 0.09 | 0.07 | 0.94 | 0.95 | 0.37 | 0.43 | 0.99 | 0.14 |
| Video: Emotional > Neutral mPFC | | | | | | | | | |
| Quadratic |  | 0.13 | 0.10 | 0.92 | 0.94 | 0.99 | 0.98 | 0.22 | 0.86 |
| Linear |  | 0.09 | 0.07 | 0.94 | 0.96 | 0.01 | 0.02 | 0.78 | 0.14 |
| *Notes.* All models include interaction terms of the independent variable with self-blame and rumination respectively; green indicates better performance of the quadratic versus linear model fit; orange indicates better performance of the linear versus quadratic model fit; abbreviations: AIC = Akaike's Information Criterion; AICc: Second-order (or small sample) Akaike's Information Criterion with a correction for small sample sizes; BIC = Bayesian Information Criterion; RMSE = Root Mean Squared Error; Sigma = Residual Standard Deviation; IFG = Inferior Frontal Gyrus; ACC = Anterior Cingulate Cortex; mPFC = medial Prefrontal Cortex; ^a^ weights. | | | | | | | | | |

| Table S10  Negative Affect Models with ToM Measures as Independent Variables | | | | | | | | | |
| --- | --- | --- | --- | --- | --- | --- | --- | --- | --- |
| Model Fit |  | **R^2^** | **R^2^ adj.** | **RMSE** | **Sigma** | **AIC^a^** | **AICc^a^** | **BIC^a^** | **Performance Score** |
| IRI Perspective Taking | | | | | | | | | |
| Quadratic |  | 0.14 | 0.11 | 0.92 | 0.94 | 0.25 | 0.21 | 0.00 | 0.57 |
| Linear |  | 0.12 | 0.10 | 0.92 | 0.94 | 0.75 | 0.79 | 1.00 | 0.43 |
| EmpaToM ToM Performance | | | | | | | | | |
| Linear |  | 0.09 | 0.06 | 0.94 | 0.96 | 0.91 | 0.93 | 1.00 | 0.71 |
| Quadratic |  | 0.09 | 0.06 | 0.94 | 0.96 | 0.09 | 0.07 | 0.00 | 0.29 |
| Video: ToM > nToM IFG | | | | | | | | | |
| Quadratic |  | 0.10 | 0.07 | 0.93 | 0.95 | 0.25 | 0.21 | 0.00 | 0.57 |
| Linear |  | 0.09 | 0.07 | 0.94 | 0.95 | 0.75 | 0.79 | 1.00 | 0.43 |
| Question: ToM > nToM IFG | | | | | | | | | |
| Linear |  | 0.08 | 0.06 | 0.95 | 0.96 | 0.81 | 0.84 | 1.00 | 0.71 |
| Quadratic |  | 0.09 | 0.06 | 0.94 | 0.96 | 0.19 | 0.16 | 0.00 | 0.29 |
| Video: ToM > nToM Temporal Gyrus | | | | | | | | | |
| Quadratic |  | 0.10 | 0.07 | 0.93 | 0.95 | 0.34 | 0.29 | 0.00 | 0.57 |
| Linear |  | 0.09 | 0.07 | 0.94 | 0.96 | 0.66 | 0.71 | 1.00 | 0.43 |
| Question: ToM > nToM Temporal Gyrus | | | | | | | | | |
| Quadratic |  | 0.12 | 0.09 | 0.93 | 0.94 | 0.84 | 0.81 | 0.02 | 0.86 |
| Linear |  | 0.09 | 0.07 | 0.94 | 0.95 | 0.16 | 0.19 | 0.98 | 0.14 |
| Video: ToM > nToM mPFC | | | | | | | | | |
| Linear |  | 0.09 | 0.07 | 0.94 | 0.96 | 0.82 | 0.85 | 1.00 | 0.71 |
| Quadratic |  | 0.10 | 0.07 | 0.94 | 0.96 | 0.18 | 0.15 | 0.00 | 0.29 |
| Question: ToM > nToM mPFC | | | | | | | | | |
| Linear |  | 0.09 | 0.07 | 0.94 | 0.95 | 0.91 | 0.92 | 1.00 | 0.71 |
| Quadratic |  | 0.10 | 0.06 | 0.94 | 0.96 | 0.09 | 0.08 | 0.00 | 0.29 |
| *Notes.* All models include interaction terms of the independent variable with self-blame and rumination respectively; green indicates better performance of the quadratic versus linear model fit; orange indicates better performance of the linear versus quadratic model fit; abbreviations: AIC = Akaike's Information Criterion; AICc: Second-order (or small sample) Akaike's Information Criterion with a correction for small sample sizes; BIC = Bayesian Information Criterion; RMSE = Root Mean Squared Error; Sigma = Residual Standard Deviation; IFG = Inferior Frontal Gyrus; mPFC = medial Prefrontal Cortex; ^a^ weights. | | | | | | | | | |

# Results from regression models without interaction terms

| S11  Regression models without interaction terms and with personal distress as independent variable | | | | | | |
| --- | --- | --- | --- | --- | --- | --- |
|  | **Outcome 1: Stress** | | | **Outcome 2: Negative affect** | | |
| *Predictors* | *ß* | *95% CI* | *p*_adj_ | *ß* | *95% CI* | *p*_adj_ |
| (Intercept) | -0.02 | -0.18 – 0.14 | 0.868 | -0.13 | -0.29 – 0.03 | 0.150 |
| Age | -0.11 | -0.33 – 0.11 | 0.578 | -0.33 | -0.55 – -0.12 | **0.009** |
| Gender [male] | 0.11 | -0.12 – 0.34 | 0.578 | 0.20 | -0.02 – 0.43 | 0.136 |
| Rumination | 0.02 | -0.21 – 0.25 | 0.868 | 0.13 | -0.11 – 0.36 | 0.334 |
| Self-blame | 0.21 | -0.02 – 0.44 | 0.275 | 0.23 | 0.00 – 0.46 | 0.108 |
| Personal distress | 0.57 | 0.35 – 0.80 | **<0.001** | 0.52 | 0.29 – 0.74 | **<0.001** |
| (Personal distress)^2^ | -0.11 | -0.43 – 0.21 | 0.712 | 0.11 | -0.21 – 0.43 | 0.490 |
| Observations | 296 | | | 296 | | |
| R^2^ / R^2^ adjusted | 0.110 / 0.091 | | | 0.146 / 0.128 | | |
| *Note.* Personal distress was measured with the Interpersonal Reactivity Index. | | | | | | |

| S12  Regression models without interaction terms and with perspective taking as independent variable | | | | | | |
| --- | --- | --- | --- | --- | --- | --- |
|  | **Outcome 1: Stress** | | | **Outcome 2: Negative affect** | | |
| *Predictors* | *ß* | *95% CI* | *p*_adj_ | *ß* | *95% CI* | *p*_adj_ |
| (Intercept) | -0.07 | -0.24 – 0.10 | 0.611 | -0.13 | -0.30 – 0.03 | 0.115 |
| Age | -0.12 | -0.35 – 0.10 | 0.480 | -0.34 | -0.56 – -0.12 | **0.002** |
| Gender [male] | -0.01 | -0.24 – 0.22 | 0.934 | 0.10 | -0.12 – 0.32 | 0.388 |
| Rumination | 0.05 | -0.19 – 0.29 | 0.788 | 0.17 | -0.06 – 0.41 | 0.153 |
| Self-blame | 0.30 | 0.06 – 0.53 | **0.046** | 0.33 | 0.10 – 0.56 | **0.005** |
| Perspective taking | -0.39 | -0.62 – -0.17 | **0.005** | -0.43 | -0.65 – -0.21 | **<0.001** |
| (Perspective taking)^2^ | 0.29 | -0.03 – 0.62 | 0.174 | 0.29 | -0.03 – 0.60 | 0.074 |
| Observations | 296 | | | 296 | | |
| R^2^ / R^2^ adjusted | 0.075 / 0.056 | | | 0.134 / 0.116 | | |
| *Note.* Perspective taking was measured with the Interpersonal Reactivity Index. | | | | | | |

# References

Berretz, G., Packheiser, J., Kumsta, R., Wolf, O. T., & Ocklenburg, S. (2021). The brain under stress-A systematic review and activation likelihood estimation meta-analysis of changes in BOLD signal associated with acute stress exposure. *Neuroscience and Biobehavioral Reviews*, *124*, 89–99. <https://doi.org/10.1016/j.neubiorev.2021.01.001>

Bzdok, D., Schilbach, L., Vogeley, K., Schneider, K., Laird, A. R., Langner, R., & Eickhoff, S. B. (2012). Parsing the neural correlates of moral cognition: ALE meta-analysis on morality, theory of mind, and empathy. *Brain Structure & Function*, *217*(4), 783–796. <https://doi.org/10.1007/s00429-012-0380-y>

Cohen, S., Kamarck, T., & Mermelstein, R. (1983). A Global Measure of Perceived Stress. *Journal of Health and Social Behavior*, *24*(4), 385. <https://doi.org/10.2307/2136404>

Faul, F., Erdfelder, E., Lang, A.-G., & Buchner, A. (2007). G* Power 3: A flexible statistical power analysis program for the social, behavioral, and biomedical sciences. *Behavior Research Methods*, *39*(2), 175–191.

Kanske, P., Böckler, A., Trautwein, F.-M., & Singer, T. (2015). Dissecting the social brain: Introducing the EmpaToM to reveal distinct neural networks and brain-behavior relations for empathy and Theory of Mind. *NeuroImage*, *122*, 6–19. <https://doi.org/10.1016/j.neuroimage.2015.07.082>

Krohne, H. W., Egloff, B., Kohlmann, C. W., & Tausch, A. (1996). Investigations with a German version of the positive and negative affect schedule (PANAS). *Diagnostica*, *42*(2), 139–156.

Lindquist, K. A., Satpute, A. B., Wager, T. D., Weber, J., & Barrett, L. F. (2016). The Brain Basis of Positive and Negative Affect: Evidence from a Meta-Analysis of the Human Neuroimaging Literature. *Cerebral Cortex*, *26*(5), 1910–1922. <https://doi.org/10.1093/cercor/bhv001>

Lorah, J. A. (2020). Interpretation of main effects in the presence of non-significant interaction effects. *The Quantitative Methods for Psychology*, *16*(1), 33–45. <https://doi.org/10.20982/tqmp.16.1.p033>

Tholen, M. G., Trautwein, F.-M., Böckler, A., Singer, T., & Kanske, P. (2020). Functional magnetic resonance imaging (fMRI) item analysis of empathy and theory of mind. *Human Brain Mapping, 41*(10), 2611–2628. <https://doi.org/10.1002/hbm.24966>

Trautwein, F.-M., Kanske, P., Böckler, A., & Singer, T. (2020). Differential benefits of mental training types for attention, compassion, and theory of mind. *Cognition*, *194*, 104039. <https://doi.org/10.1016/j.cognition.2019.104039>
